# PD-L1-CD80 interactions are required for intracellular signaling necessary for dendritic cell migration

**DOI:** 10.1126/sciadv.adt3044

**Published:** 2025-01-29

**Authors:** Uma Kantheti, Tadg S. Forward, Erin D. Lucas, Johnathon B. Schafer, Pierce J. Tamburini, Matthew A. Burchill, Beth Ann Jirón Tamburini

**Affiliations:** ^1^ Department of Medicine, Division of Gastroenterology and Hepatology, University of Colorado School of Medicine, Aurora, CO, USA.; ^2^ Immunology Graduate Program, University of Colorado School of Medicine, Aurora, CO, USA.; ^3^ Medical Scientist Training Program, University of Colorado School of Medicine, Aurora, CO, USA.; ^4^ Department of Immunology and Microbiology, University of Colorado School of Medicine, Aurora, CO, USA.

## Abstract

Programmed cell death protein 1 (PD-1) and programmed death ligand 1 (PD-L1) interactions are targets for immunotherapies aimed to reinvigorate T cell function. Recently, it was documented that PD-L1 regulates dendritic cell (DC) migration through intracellular signaling events. In this study, we find that both preclinical murine and clinically available human PD-L1 antibodies limit DC migration. We show that cis interactions between PD-L1 and CD80 are critical for promoting migration and define specific regions within these proteins necessary for migration. Furthermore, we demonstrate that αPD-L1 significantly impedes DC migration in a B16 melanoma tumor model. Last, we outline how blocking cis PD-L1:CD80 interactions or mutation of the intracellular domain of PD-L1, in an imiquimod-induced murine model of psoriasis, limits DC migration to the lymph node, decreases interleukin-17 production by CD4^+^ T cells in the lymph node, and reduces epidermal thickening. Therefore, PD-L1 and CD80 interactions are important regulators of DC migration to the draining lymph node.

## Teaser

Blocking PD-L1:CD80 interactions prevents DC migration and limits T_H_17 responses during imiquimod induced psoriasis.

## INTRODUCTION

Interactions between programmed death ligand 1 (PD-L1) and its receptor programmed cell death protein 1 (PD-1) result in inhibition of T cell receptor signaling and subsequent T cell responses (*1*, *2*). However, recent evidence has demonstrated that PD-L1 is a bifunctional molecule that also participates in ligand-ligand interactions with costimulatory ligand CD80 (*3*–*6*). Both PD-L1 and B7.1 (CD80) are members of the B7 ligand family, which are structurally homologous cell-surface protein ligands that are expressed on antigen-presenting cells (APCs) (*7*). Dendritic cell (DC) activation leads to up-regulation of both PD-L1 and CD80, and coexpression allows for cis binding between PD-L1 and CD80 and prevents trans binding between PD-L1 and PD-1 (*5*, *6*, *8*). In the absence of PD-L1, CD80 forms homodimers, which bind to CTLA4 in trans, leading to removal of CD80 via trans-endocytosis (*6*, *9*, *10*). The interaction between CD80 and PD-L1 is favored because the affinity of this interaction is higher compared to CD80 homodimers (*6*). The cis PD-L1:CD80 interaction enables activation of naïve T cells through CD80:CD28 binding while simultaneously preventing inhibitory PD-L1:PD-1 interactions (*5*, *6*). Upon limiting amounts of CD80, such as in the absence of proper activation, PD-L1 monomer is available for interaction with PD-1 (*6*, *11*). These different interactions between costimulatory and coinhibitory molecules between APCs and T cells emphasize the intricate mechanisms that control T cell activation and deactivation. Furthermore, these data outline the importance of equal stoichiometries of both PD-L1 and CD80 to promote the cis duplex on activated APCs for optimal T cell responses (*5*, *8*).

Conventional DCs (cDCs), termed “professional APCs” populate peripheral tissues such as the skin, mucosal surfaces, and most solid organs. cDCs are therefore positioned to allow for efficient antigen uptake and detection of pathogens (*12*, *13*). Monocytes traffic from the blood to inflamed tissue via CCR2, where they differentiate into monocyte-derived DCs (moDCs) (*13*). Upon pathogen recognition, DCs undergo an activation process resulting in increased cellular motility and surface expression of cell surface proteins [major histocompatibility complex (MHC) class II, CD80/86, CC chemokine receptor 7 (CCR7), and PD-L1] to promote efficient transport and presentation of antigens (*14*–*16*). The up-regulation of CCR7 by migratory DCs mediates intravasation through the button-like junctions of CCL21-expressing lymphatic capillaries (*17*–*20*). Upon entry through so called “transmigratory cups,” DCs enter the lumen of the lymphatic capillaries and crawl toward the draining lymph node (dLN) via a CCL21 gradient (*18*, *21*). Once in the lymphatic vessel, the DCs are propelled by laminar flow resulting from lymphatic muscle cell pumping in the vessels (*21*, *22*). DCs land in the subcapsular sinus of the dLN where further positioning is regulated by expression of CCL19 and CCL21 by fibroblastic reticular cells in the cortex of the dLN (*23*). Migratory XCR1^+^ cDC1s travel the furthest into the dLN because of high levels of membrane-associated CCR7, which guide them deep into the paracortex, where CD8^+^ T cells reside (*19*, *22*, *24*). CD11b^+^IRF4^+^ migratory cDC2s localize within follicular areas of LN where CD4^+^ T cells reside (*22*). Despite the breadth of knowledge regarding chemokine-mediated migration of DCs, open questions remain regarding the contribution of B7 ligands such as PD-L1 and CD80 to DC migration.

PD-L1 and CD80 both have extracellular domains that are structurally related to variable regions of immunoglobulins (*25*) and have short cytoplasmic tails that have been shown to serve as sites for intracellular signaling within DCs (*26*, *27*). While the cell-extrinsic effects of cis PD-L1:CD80 interactions on T cell immunity have been well characterized, the cell-intrinsic properties within migratory DCs remain understudied. We previously characterized a signaling motif within the cytoplasmic tail of PD-L1 that promoted dermal DC migration to the dLN upon polyI:C immunization and cutaneous *Listeria monocytogenes* infection in mice (*26*). The cell-intrinsic signals through PD-L1 were essential for CCR7-mediated DC migration as downstream G-alpha subunit activation and F-actin polymerization were decreased when intracellular PD-L1 residues (amino acids 277 to 279) were changed to alanine, termed *Pdl1^CyMt^* (*26*). Further characterization of *Pdl1^CyMt^* revealed STAT3 and paxillin bound to PD-L1, both of which have been previously shown to be critical in focal adhesions and cell motility (*28*, *29*). However, it was not determined how extracellular interactions with receptors or clinically relevant PD-L1 antibodies might affect this PD-L1 intracellular signaling.

In this study, we used in vivo and in vitro approaches to demonstrate that PD-L1 engagement with CD80 in cis on migratory DCs is necessary for migration to the LN during cutaneous inflammation. We provide evidence that the amino acid residues necessary for CD80:PD-L1 interactions to promote DC migration include tyrosine-56 on PD-L1, and leucine-107 on CD80, as well as the intracellular tail of PD-L1. All clinically available antibodies either covered or sterically limited the necessary residues for PD-L1:CD80 extracellular interactions, the consequence of which was reduced DC migration. Further, administration of anti–PD-L1 atezolizumab caused reduced DC migration in a murine melanoma tumor model. Last, we outline how preventing PD-L1:CD80 interactions or mutating the PD-L1 cytoplasmic domain in a model of psoriasis limits DC migration, interleukin-17 (IL-17) production by CD4^+^ T cells, and epidermal thickening associated with disease. Together, this manuscript provides an undescribed function for cis PD-L1 and CD80 interactions within DCs. These findings provide rationale for defining the exact mechanisms of PD-L1 signaling and may be an explanation as to why PD-L1 antibodies may not be as effective as PD-1 antibodies in a cancer setting (*30*) but may be a potential target for autoimmune inflammatory disorders like psoriasis where DC migration is detrimental (*31*).

## RESULTS

### PD-L1 ligation with atezolizumab, durvalumab, and avelumab prohibits efficient chemokine-mediated migration of human monocyte–derived DCs

**Fig. 1.**
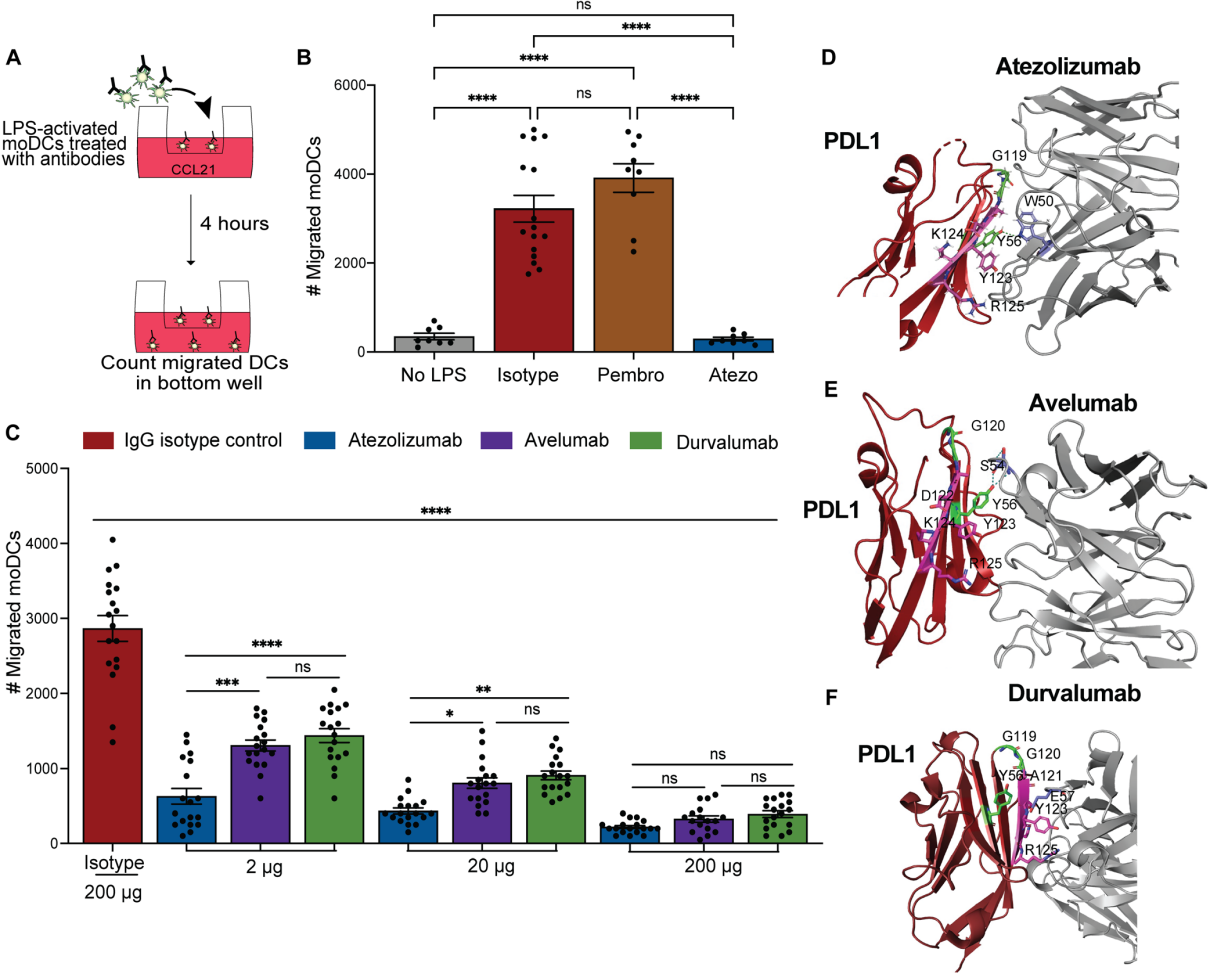
Human antibodies directed at PD-L1 and PD-1 have different effects on moDC migration. (**A**) Experimental scheme for (C) and (B). (**B**) Number of migrated moDCs after 4 hours of LPS treatment (200 ng/ml) and IgG isotype control, pembrolizumab (αPD-1 antibody) or atezolizumab (αPD-L1 antibody) at 200 μg/ml. Data shown in figure are data from four independent experiments with two to four replicates per treatment group. (**C**) Number of migrated moDCs after 4 hours of LPS treatment (200 ng/ml) with IgG isotype control, atezolizumab, avelumab, and durvalumab at indicated concentrations from 2, 20, or 200 μg/ml as described in experimental scheme in (A). (**D**) Crystal structure of the extracellular domain of human PD-L1 interacting with the heavy chain of atezolizumab depicting the amino acid residues in contact. Green and magenta areas on PD-L1 indicate the CD80 and PD-1–binding domains, respectively. Tryptophan 50 depicted in blue within atezolizumab depicts the interacting residue with PD-L1 (*50*, *51*). (**E**) Crystal structure of the extracellular domain of human PD-L1 interacting with the heavy chain of avelumab depicting the amino acid residues in contact. Green and magenta areas on PD-L1 indicate the CD80 and PD-1–binding domains, respectively. Serine-54 depicted in blue within avelumab depicts the interacting residue with PD-L1 (*34*, *50*). (**F**) Crystal structure of the extracellular domain of human PD-L1 interacting with the heavy chain of durvalumab depicting the amino acid residues in contact. Green and magenta areas on PD-L1 indicate the CD80 and PD-1 binding domains, respectively. Glutamic acid 57 depicted in blue within durvalumab depicts the interacting residue with PD-L1 (*52*). Experiment was repeated four times with three to six technical replicates per experiment. Statistical significance was determined using one-way analysis of variance (ANOVA) with multiple comparisons. Every treatment group was statistically significantly different (*P* < 0.0001) than the isotype control. *P < 0.05, **P < 0.01, ***P < 0.001, ****P < 0.0001; ns, *P* > 0.05. Error bars indicate SEM.

In our prior work, we defined an important mechanism by which PD-L1 expression by DCs is required for effective intracellular signals during normal chemokine-mediated migration (*26*). We outlined the importance of three intracellular residues within *Pdl1^CyMt^* mice to promote chemokine-mediated migration and drive T cell programming when migration of DCs was required in a genetically manipulated animal model (*26*). However, it had yet to be determined whether PD-L1 extracellular interactions, with either PD-1 or CD80 (*5*), participate in the regulation of DC migration or if the same PD-L1 regulation of DC migration is conserved in human DCs. The molecular interactions between PD-L1 and PD-1 visualized using the available crystal structures from Protein Data Bank (PDB) demonstrates partially overlapping binding sites between PD-L1 with PD-1 and its other binding partner CD80 (*32*, *33*). We sought to address questions related to necessary PD-L1–binding partners in human DC migration using clinically available antibodies that prohibit PD-L1 extracellular interactions by using either atezolizumab (αPD-L1) or pembrolizumab (αPD-1). To address this, we differentiated human peripheral blood mononuclear cells (PBMCs) that were CD14^+^ into moDCs using IL-4 and granulocyte-macrophage colony-stimulating factor (GM-CSF) (Fig. 1A and fig. S1A). Consistent with prior studies, we observed chemokine-mediated DC migration upon activation with lipopolysaccharide (LPS) (Fig. 1B) (*34*). We tested whether blocking the ability of PD-L1 to bind to PD-1 on the surface of the DCs using αPD-1 pembrolizumab would impede DC migration (Fig. 1B). We found no defect in DC migration in the presence of αPD-1 pembrolizumab, suggesting that the interaction between PD-1 and PD-L1 in cis (*35*) did not regulate DC migration (Fig. 1B). However, there was a notable difference in chemokine-mediated DC migration when the PD-L1 antibody, atezolizumab, was added to the DCs (Fig. 1B). PD-L1 (fig. S1B), CD80 (fig. S1C), and PD-1 (fig. S1D) are up-regulated following LPS activation on the human blood–derived DCs. We saw loss of surface-staining of PD-L1 by flow cytometry in the presence of atezolizumab, which we did not observe with isotype or pembrolizumab (fig. S1B). These findings indicate that atezolizumab blocked the binding of the fluorescently labeled PD-L1 antibody (fig. S1B). We did not see loss of PD-1 staining by flow cytometry after pembrolizumab, suggesting that PD-1 expression on DCs is negligible despite a slight, but not significant, increase compared to no LPS and the fluorescence minus one stain (fig. S1, D and E). Like our findings in murine models where PD-L1 is absent or the cytoplasmic domain is mutated, we found no difference in CCR7 or CD86 expression between isotype, pembrolizumab, or atezolizumab (fig. S1, F and G) (*26*). Together, these findings show that moDCs derived from human blood have a PD-L1–dependent migratory function.

Two critical properties of the clinically available PD-L1 antibodies include (i) binding affinity and (ii) the amino acid residues that are masked by the antibody. To date, there are three Food and Drug Administration–approved PD-L1 antibodies: atezolizumab, durvalumab, and avelumab. The crystallized structures in complex with PD-L1 and the binding kinetics of these antibodies have been well described. The binding affinities based on the dissociation constant (*K*_d_) are similar between atezolizumab and durvalumab where *K*_d_ = 1.75 and 0.667 nM, respectively (*36*). Alternatively, avelumab’s binding affinity to PD-L1 is approximately 10-fold stronger with a *K*_d_ = 0.0467 nM, a critical distinction in binding kinetics compared to avelumab and durvalumab (*36*). Given our results in Fig. 1B showing a marked decrease in moDC migration after atezolizumab treatment, we next asked whether a lower *K*_d_ may have a greater effect on DC migration. Instead, we found that the most notable decrease in DC migration was caused by the antibody with the highest *K*_d_ (lower affinity), atezolizumab, when compared to durvalumab and avelumab at the lowest dose tested (2 μg) (Fig. 1C). This was consistent at an intermediate dose of 20 μg/ml where both durvalumab and avelumab had markedly more DCs migrated compared to atezolizumab (Fig. 1C). However, at the highest given dose of 200 μg/ml, all antibodies prevented DC migration to a similar extent, consistent with full saturation of the available ligand, PD-L1, on activated moDCs (Fig. 1C). Moreover, different dose titrations for atezolizumab had a negligible effect overall on DC migration, unlike avelumab and durvalumab where lower dosages permitted more DCs to migrate across an 8-μm membrane although still much less compared to the isotype-treated DCs (Fig. 1C).

Since our data emphasized that binding affinity of antibodies to PD-L1 does not affect moDC migration to the extent that we predicted, we next asked whether the amino acids masked by the associated PD-L1 antibodies were different and may explain the differences in moDC migration. The mapping of the PD-L1 residues within the human extracellular immunoglobulin V (IgV)–like domain from published crystal structures revealed the distinct but slightly overlapping surfaces where CD80 and PD-1 associate with PD-L1 (*32*, *37*). Prior studies identified several key amino acid residues necessary for facilitation of binding with PD-1 such as PD-L1 residues A121, D122, Y123, K124, and R125, with the most critical residues being D122 and Y123 (*32*, *33*). The residues most essential for PD-L1 binding with CD80 are PD-L1 residues Y56, G119, and G120 (*32*). Since others have established that association of PD-L1 with CD80 is facilitated through tyrosine-56 on PD-L1 and leucine-107 on CD80 (*3*–*6*), we further predicted that there was a correlation between the proximity by which atezolizumab, avelumab, and durvalumab bind near tyrosine-56 and the magnitude by which DC migration would be affected. Upon examination of the binding characteristics of each PD-L1 antibody, we observed that atezolizumab covered the most residues in the CD80 binding domain of PD-L1 and also formed a polar interaction with tyrosine-56 on PD-L1 with tryptophan 50 within the heavy chain (Fig. 1D) (*38*–*40*). Avelumab also formed a biochemical interaction with tyrosine-56 although not G119 or G120 (Fig. 1E) (*37*, *38*). Durvalumab did not mask any of the known the PD-L1 residues necessary for CD80 binding and instead associated with D122 and Y123 on PD-L1, both which of are crucial for PD-1 binding (Fig. 1F) (*37*, *38*). However, durvalumab still limits CD80 binding based on prior reports (*36*). These findings further solidify a molecular basis for the unique effects atezolizumab, avelumab, and durvalumab have on DC migration and demonstrate the importance of the availability of the CD80-binding domain to engage with PD-L1 on activated DCs to promote migration. Last, these data suggest that clinically available PD-L1 antibodies elicit unique and specific effects based on the residues they interact with and suggest the importance of dosing and clinical outcomes with these different antibodies.

### Dermal DC migration requires PD-L1 extracellular interactions and CD80/86 expression in vivo

**Fig. 2.**
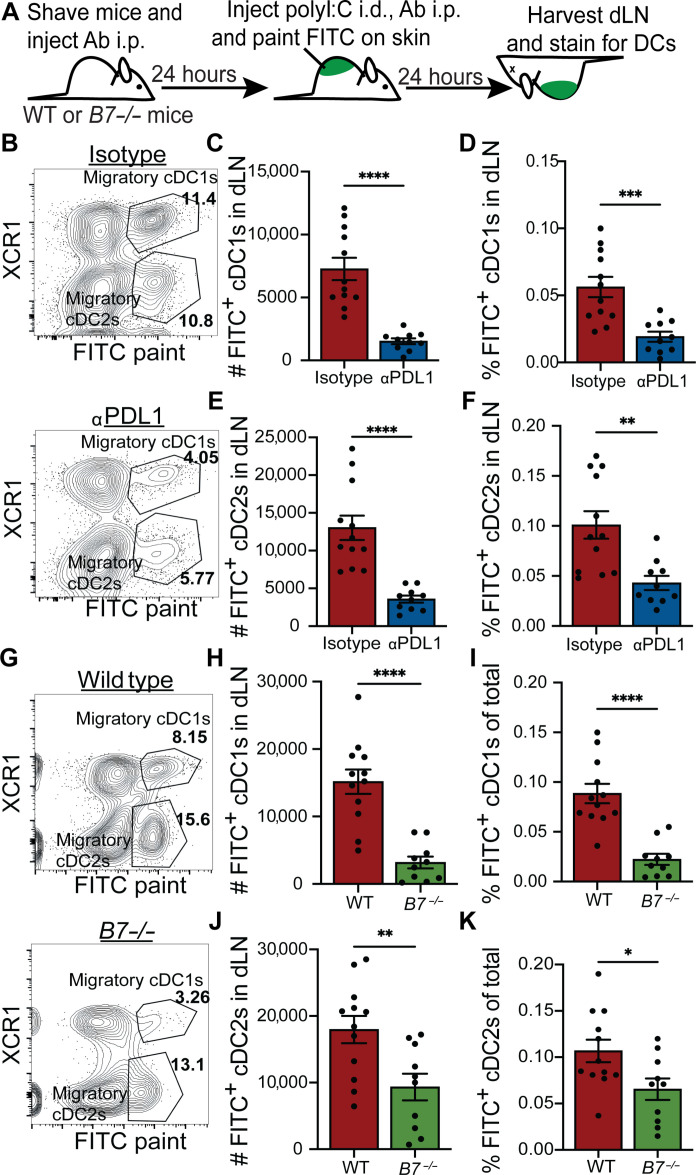
PD-L1 antibody and loss of B71/2 diminishes dermal DC migration in mice after polyI:C immunization. (**A**) Experimental scheme for (B) to (K). (**B**) Representative flow plots showing in the dLN 24 hours after polyI:C injection and FITC paint application. Gated on live, MHC class II high, CD11c high, and FITC^+^ skin-derived cDC subsets in isotype and αPD-L1 (10F9.G2 clone)–treated mice. (**C**) Number of FITC^+^ cDC1s (XCR1^+^CD11b^−^) in the dLN 24 hours after intradermal polyI:C injection and topical FITC paint application. (**D**) Percentage of FITC^+^ cDC1s of total cells in the dLN. (**E**) Number of FITC^+^ cDC2s (CD11b^+^XCR1^−^) in the dLN 24 hours after intradermal polyI:C injection and topical FITC paint application. (**F**) Percentage of FITC^+^ cDC2s of total cells in the dLN. (**G**) Representative flow plots showing FITC^+^ skin-derived cDC subsets in WT and *B7^−/−^* mice. Gated on live, MHC class II high, and CD11c. (**H**) Number of FITC^+^ cDC1s the dLN 24 hours after intradermal polyI:C injection and topical FITC paint application. (**I**) Percentage of FITC^+^ cDC1s of total cells in the dLN. (**J**) Number of FITC^+^ cDC2s in the dLN 24 hours after intradermal polyI:C injection and topical FITC paint application. (**K**) Percentage of FITC^+^ cDC2s of total cells in the dLN. Data shown in figure represent two combined experiments. Each dot represents one LN where at least two mice per group were used. Experiment was completed on three to five separate occasions with two to three mice per treatment group. Statistical significance was determined using Student’s *t* test. *P < 0.05, **P < 0.01, ***P < 0.001, ****P < 0.0001; ns, *P* > 0.05. Error bars indicate SEM; i.p., intraperitoneal.

We next sought to determine whether CD80 or a murine anti–PD-L1 clone that blocks both PD-L1:PD1 and PD-L1:CD80 interactions were important to facilitate DC migration from the murine skin to the associated dLN. To this end, we evaluated in vivo cellular trafficking of skin-derived cDCs through utilization of a fluorescein isothiocyanate (FITC) skin painting assay where FITC acquired by dermal DCs that migrate to the dLN can be used to distinguish between migratory and LN-resident DCs (Fig. 2A and fig. S2A) (*26*, *41*, *42*). We used the anti-mouse PD-L1 10F9.G2 antibody clone due to similarities in PD-L1 binding to anti-human atezolizumab (*6*) to confirm, in vivo, that DC migration is disrupted in the presence of an antibody that blocks PD-1 and CD80 interactions with PD-L1. After intraperitoneal injection of IgG isotype or PD-L1 blocking antibodies as well as intradermal injection of polyI:C (Fig. 2A), we found both a lower frequency and number of FITC^+^ DCs and in the skin-dLNs in mice treated with the 10F9.G2 αPD-L1 antibody, but not an isotype control antibody (fig. S2B). Similarly, we observed fewer FITC^+^ cDC1s (Fig. 2, B to D) and cDC2s (Fig. 2, B, E, and F) in the dLN by number and frequency, and both FITC^+^ cDC1s and cDC2s had decreased PD-L1 expression, by geometric mean fluorescence intensity (gMFI), when compared to isotype-treated mice (fig. S2, C to H). Consistent with other reports that loss of PD-L1 interactions with CD80 resulted in CD80 homodimerization and removal by CTLA-4–mediated trans-endocytosis, on migratory cDCs in vivo (*6*, *9*, *26*, *43*), we found decreased surface expression of CD80 by gMFI on FITC^+^ cDC1s and cDC2s (fig. S2, C to H). However, we cannot differentiate between loss of CD80 expression and loss of staining caused by the PD-L1 antibody blocking CD80 antibody binding.

**Fig. 3.**
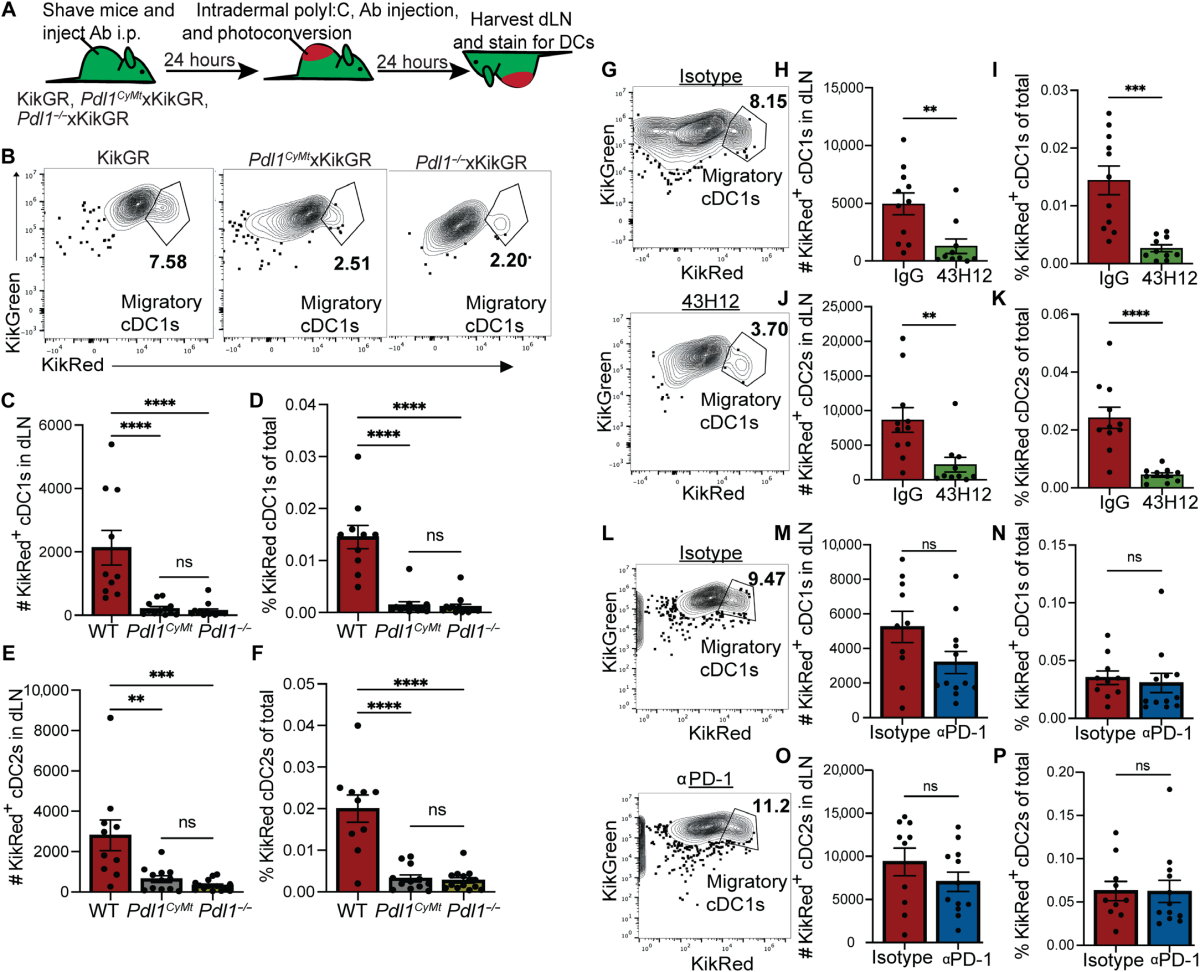
Interactions between PD-L1 and CD80 regulate dermal DC migration in KikGR mice. (**A**) Experimental KikGR scheme for (B) to (P). (**B**) Representative flow plots showing KikRed^+^ (photoconverted) skin-derived cDC subsets in KikGR mice crossed to *Pdl1^CyMt^* or *Pdl1^−/−^*. Gated on CD45^+^, MHC class high, CD11c high, and XCR1^+^ for cDC1s and CD11b^+^ for cDC2s. (**C** and **D**) Number and percentage of KikRed^+^ cDC1s (XCR1^+^CD11b^−^) in the dLN 24 hours after intradermal polyI:C injection and photoconversion in indicated strain. (**E** and **F**) Number and percentage of KikRed^+^ cDC2s (CD11b^+^XCR1^−^) in the dLN 24 hours after intradermal polyI:C injection and photoconversion in indicated strain. (**G**) Representative flow plots showing KikRed^+^ (photoconverted) skin-derived cDC subsets in isotype and 43H12 antibody–treated mice. (**H** and **I**) Number and percentage of KikRed^+^ cDC1s in the dLN 24 hours after intradermal polyI:C injection and photoconversion in isotype or PD-L1 43H12 antibody. (**J** and **K**) Number and percentage of KikRed^+^ cDC2s in the dLN 24 hours after intradermal polyI:C injection and photoconversion in isotype or PD-L1 43H12 antibody. (**L**) Representative flow plots showing KikRed^+^ (photoconverted) skin-derived cDC subsets in isotype and αPD-1–treated mice. (**M** and **N**) Number and percentage of KikRed^+^ cDC1s in the dLN 24 hours after intradermal polyI:C injection and violet photoconversion in isotype and PD-1 antibody–treated mice. (**O** and **P**) Number and percentage of KikRed^+^ cDC2s in the dLN 24 hours after intradermal polyI:C injection violet photoconversion in isotype and PD-1 antibody–treated mice. Data shown are from two experiments performed on separate occasions with two to three mice per condition and each dot represents one LN. The same experiment was completed three times with two to three mice per treatment group. Statistical significance was determined using Student’s *t* test. **P < 0.01, ****P < 0.0001; ns, *P* > 0.05. Error bars indicate SEM.

Since the blockade of PD-L1 through the 10F.9G2 clone results in loss of CD80 interactions, and potentially the physical removal of CD80 from the surface of migratory DCs, including cDC1s and cDC2 (fig. S2, C to H) (*9*, *43*), we next evaluated whether CD80 itself was important for dermal DC migration. Using *B7^−/−^* knockout mice that lack both B7.1 and B7.2 (CD80 and CD86), we observed considerably less total FITC^+^ DCs, cDC1s, and cDC2s in the dLNs in *B7^−/−^* mice compared to wild-type (WT) mice after polyI:C injection (Fig. 2, G to K, and fig. S2I). Consistent with the established genotype of *B7^−/−^* mice, there was no surface-associated CD80 detected and, interestingly, lower levels of PD-L1 by gMFI on FITC^+^ cDC1s and cDC2s in *B7^−/−^* mice compared to WT (fig. S2, J to O). Topical application of FITC induces a contact hypersensitivity reaction that results in a large influx of DCs into the skin-dLNs; therefore, we moved to a less inflammatory model (*41*, *42*, *44*). We used KikGR knock-in mice where exposure to violet light causes the conversion of the Kikume green fluorescent protein to red fluorescence and does not induce contact hypersensitivity (Fig. 3A) (*45*, *46*). Since *B7^−/−^* mice lack both CD80 and CD86, we crossed KikGR mice with *Cd80^−/−^* mice to interrogate the role of CD80 alone in dermal DC migration (fig. S3A). As with *B7^−/−^* mice, we saw fewer KikRed^+^ DCs in the dLN regardless of the subset evaluated (fig. S3). We confirmed the loss of CD80 expression, which also resulted in a decrease in PD-L1 levels (gMFI) (fig. S3). These findings show that antibodies that block PD-L1 extracellular interactions or loss of B7.1 or B7.2 expression on migratory DCs results in less DC migration from the skin to the dLN in the setting of local dermal inflammation.

### Antibody blockade of PD-L1:CD80 interactions but not PD-L1:PD-1 regulates DC movement from the skin to dLN

We next evaluated PD-L1 10F9.G2 antibody in the KikGR model. Skin-derived DCs were identified in the dLN as CD11c^+^MHC class II^high^ cells that were KikRed^+^ (fig. S3A). Twenty-four hours after intradermal polyI:C injection, migratory DCs from WT mice that were intraperitoneally injected with either IgG isotype control antibody or αPD-L1 10F9.G2 were assessed by flow cytometry. We found decreased DC migration with the 10F9.G2 PD-L1 antibody, similar to FITC paint, as well as decreased amounts of surface-associated PD-L1 and CD80 (figs. S2B and S4, A and B).

Having previously demonstrated reduced DC migration in the FITC paint model in *Pdl1^−/−^* and *Pdl1^CyMt^* mice (*26*), we assessed DC migration in these two strains via the kikGR model. We found decreased DC migration in both *Pdl1^−/−^* and *Pdl1^CyMt^* mice (Fig. 3, A and B, and fig. S4C) by both number and frequency of cDC1s (Fig. 3, B to D) and cDC2s (Fig. 3, E to F). Consistent with our published FITC paint data, we found equivalent expression of PD-L1 compared to WT in the *Pdl1^CyMt^* DCs (fig. S4, D to F) and reduced CD80 expression in *Pdl1^−/−^* but not *Pdl1^CyMt^* DCs (fig. S4, D to F). Having previously established that deletion of CD80 and external ligation of PD-L1 with the αPD-L1 10F9.G2 clone reduces DC migration by FITC paint and KikGR (Fig. 2 and figs. S3 and S4A), we further probed the necessity of PD-L1:CD80 interactions by using an antibody clone, PD-L1 43H12, which prohibits CD80:PD-L1 interaction while leaving PD-L1:PD-1 binding intact (*47*, *48*). Administration of PD-L1 43H12 antibodies in KikGR mice resulted in considerably less KikRed^+^DCs in the dLN 24 hours after polyI:C injection (fig. S4A) where both cDC1 and cDC2 frequency and number were reduced in the dLN following αPD-L1 43H12 administration (Fig. 3, G to K). Like the PD-L1 10F9G.2 antibody clone, the PD-L1 43H12 antibody clone resulted in lost PD-L1 staining as assessed by gMFI as a result of steric hindrance of the flow cytometry antibody with the blocking antibody (fig. S4B). Furthermore, the PD-L1 43H12 antibody clone also resulted in loss of CD80 on the surface of the DC as previously demonstrated with the PD-L1 10F9G.2 clone (figs. S4B and S2, D, F, and H) (*6*, *9*). These findings are consistent with the idea that the PD-L1 extracellular interactions with CD80 are important for the regulation of DC migration necessary following DC activation. To further establish whether PD-1 contributes to regulating DC migration in our animal model, we treated mice with αPD-1 and assessed differences in DC migration (Fig. 3L and fig. S5A). We identified KikRed^+^ cDC1s (XCR1^+^CD11b^−^) in the dLN and saw no significant differences in frequency or number of cDC1s between αPD-1– and isotype-treated mice (Fig. 3, L to N) consistent with our human DC migration assay (Fig. 1). Similarly, KikRed^+^ cDC2s (CD11b^+^XCR1^−^) migrated in comparable levels between αPD-1– and isotype-treated mice (Fig. 3, L, O, and P). PD-L1 and CD80 gMFI on KikRed^+^ cDC1s and cDC2s was unchanged across antibody treatments (fig. S5, B to D). To confirm that the comparable DC migration in αPD-1–treated mice was not because of a specific clone of αPD-1, we performed the DC migration assay using another αPD-1 clone (clone 29F.1A12). We obtained similar results with both clones of αPD-1 (fig. S5, E to F). Thus, disruption of PD-L1:PD-1 interactions by blocking PD-1 does not interfere with PD-L1–dependent dermal cDC migration in vivo.

### In vitro–differentiated DCs lacking PD-L1:CD80 interactions fail to migrate to CCL21

**Fig. 4.**
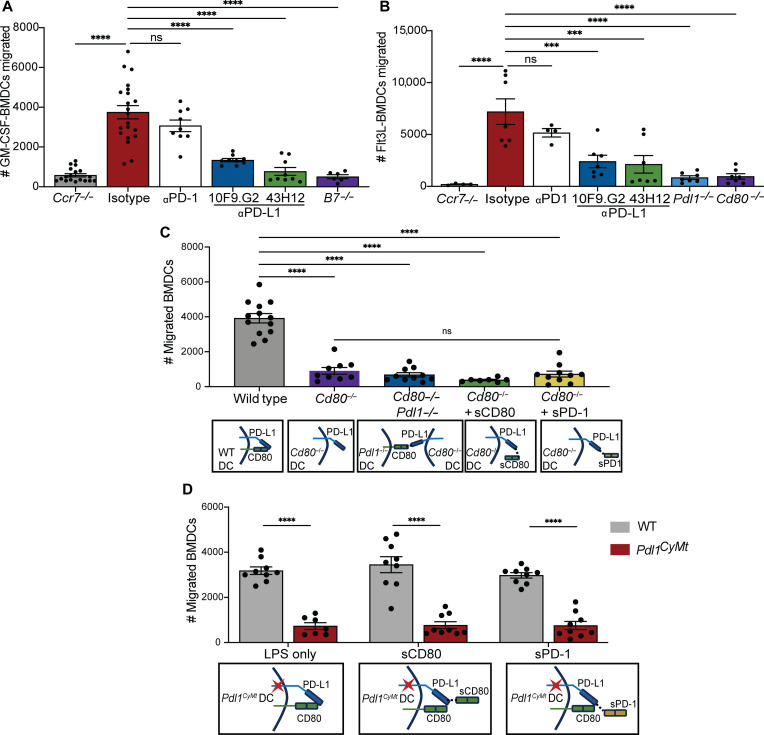
DCs fail to respond to CCL21 in the presence of αPD-L1 antibodies and require cis PD-L1:CD80 interactions. (**A**) Number of LPS-stimulated GM-CSF BMDCs (200 ng/ml) migrated across a transwell membrane to CCL21 (1 μg/ml) after 4 hours in the presence of blocking antibodies (200 μg/ml). (**B**) Number of LPS-stimulated Flt3L BMDCs (200 ng/ml) migrated across a transwell membrane to CCL21 (1 μg/ml) after 4 hours in the presence of blocking antibodies (200 μg/ml). (**C**) Number of LPS-stimulated (200 ng/ml) GM-CSF BMDCs, derived from indicated strains, migrated across a transwell to CCL21 (1 μg/ml) after 4 hours in the presence of soluble CD80 or soluble PD-1 as depicted in (A). (**D**) Number of LPS-stimulated (200 ng/ml) GM-CSF BMDCs, either WT or *Pdl1^CyMt^*, migrated across a transwell in the presence of CCL21 after 4 hours and in the presence of soluble CD80 or soluble PD-1 as depicted in (A). Data in (A) represent five combined experiments from BMDCs derived from five separate mice, and data in (B) represent two combined experiments from DCs derived from two mice. Data in (C) and (D) represent two combined experiments with BMDCs derived from two mice. Each experiment was completed two times with three to six technical replicates per treatment group. Statistical significance was determined using one-way ANOVA. ***P < 0.001, ****P < 0.0001; ns, *P* > 0.05. Error bars indicate SEM.

Thus far, we have established that PD-L1 and CD80 are required for DC migration and that blocking extracellular interactions with CD80 and not PD-1 prohibit DC migration in an in vivo murine model. We next confirmed that the differences in DC migration were specific to DC-mediated chemokine migration and not a consequence of other cell types that express these molecules. To address whether CCR7-dependent DC migration was affected in the presence of blocking PD-L1 antibodies, we used an in vitro transwell assay in which CCL21 was placed in the bottom chamber and either GM-CSF or FMS like tyrosine kinase 3 ligand (Flt3L)–derived bone marrow–derived DCs (BMDCs) were placed in the top chamber as described in Fig. 1. Isotype-treated and αPD-1–treated GM-CSF–derived BMDCs activated with LPS migrated efficiently through the 5-μm pores; however, neither αPD-L1 10F9G.2–treated WT BMDCs, αPD-L1 43H12–treated WT BMDCs, nor *B7^−/−^* BMDCs migrated more than *Ccr7^−/−^* BMDCs after LPS activation (Fig. 4A). As with human blood–derived DCs, murine GM-CSF–derived BMDCs confer a more inflammatory phenotype than cDCs naturally developing in mice (*49*–*51*). To determine whether differences in transwell migration were a result of the inflammatory phenotype specific to GM-CSF–derived BMDCs, we cultured bone marrow in the presence of Flt3L to generate BMDCs closer to what is observed in vivo for cDCs (*52*) and found a comparable defect in migration in αPD-L1 (10F9.G2 and 43H12)–treated BMDCs similar to *Ccr7^−/−^* and *Pdl1^−/−^* BMDCs (Fig. 4B) (*19*, *26*). Furthermore, to confirm that less DC migration was due to loss of only CD80 and not CD86 in *B7^−/−^* mice, we cultured *Cd80^−/−^* BMDCs that do not have the B7.2 (CD86) gene deleted (Fig. 4B and fig. S6A) (*53*). Similar to *B7^−/−^* mice and *Cd80^−/−^* mice, *Cd80^−/−^* BMDCs had reduced PD-L1 expression and CD86 expression after LPS (fig. S6A). While we believed the differences in migration to be due to limited cis PDL1:CD80 interactions, another possible explanation for less DC migration in αPD-L1–treated BMDC cultures could result from diffuse coating of the cell membrane by the antibodies, which could impair proper CCR7 ligation with the CCL21 ligand. To address this possibility, we used antibodies directed at CD45 using the same dosages as the αPD-L1 10F9.G2 and 43H12 clones and found that αCD45-treated BMDCs migrated to CCL21 at similar levels of isotype-treated BMDCs at all doses (fig. S6B). Thus, loss of migration was not globally due to antibody coating of DCs. To confirm the binding occupancy of PD-L1 in the presence of PD-L1 43H12 or 10F9.G2 antibodies, we used the fluor-conjugated anti-IgG antibodies (fig. S6C). As expected, we found increased anti-IgG–associated signal in the presence of PD-L1–blocking antibodies compared to untreated BMDCs (fig. S6C), suggesting that the ligand remained on the surface of the BMDCs. We also found little to no internalization of PD-L1 or 43H12 antibody but did detect intracellular PD-L1 antibody after 10F.G2 treatment on BMDCs (fig. S6C). Treatment of either αPD-L1 10F9.G2 or 43H12 before flow staining completely abolished staining of PD-L1^+^ BMDCs, indicating appropriate dosage with the observed binding occupancy of these antibodies on BMDCs (fig. S6D). We also found surface expression of CD80 on BMDCs after PD-L1 43H12 and 10F9.G2 antibody treatment (fig. S6D). Similar to *Pdl1^−/−^* and *Pdl1* cytoplasmic mutant DCs (*26*), αPD-L1–treated BMDCs and *CD80^−/−^* BMDCs had similar amounts of surface-associated CCR7 (fig. S6, A and E) but reduced actin polymerization (F-actin) and extracellular signal–regulated kinase (ERK) phosphorylation in response to CCL21 compared isotype-treated BMDCs (fig. S7). These findings suggest that lost CD80 binding to PD-L1 caused by antibody blockade or loss of CD80 entirely results in the same defect in downstream CCR7 signals, pERK and F-actin. These findings further build on prior published data from our laboratory demonstrating the importance of PD-L1 signaling for proper Gα subunit activation downstream of CCR7, which is necessary for cytoskeletal assembly in the context of cellular movement toward a chemokine gradient (*26*, *28*).

### Cis, not trans, interaction of CD80 with PD-L1 are necessary to promote DC migration

As other groups have suggested the possibility of PD-L1 interactions with CD80 in trans regulate PD-L1 signals (*3*, *48*), we examined whether DC migration depended exclusively on either cis or trans PD-L1:CD80 interactions. To address whether a trans interaction was sufficient to promote DC migration, we cocultured LPS activated *Pdl1^−/−^* BMDCs with *Cd80^−/−^* BMDCs (fig. S8A). Extracellular engagement of PD-L1 with CD80 in trans was not sufficient to rescue migration when compared to *Cd80^−/−^* and *Pdl1^−/−^* BMDCs (Fig. 4C). As murine BMDCs in our culture system did not express detectable PD-1 but did express membrane associated CD80 (fig. S8B), we used *Cd80^−/−^* BMDCs to determine the contributions of either sCD80 or sPD-1 to CCR7-driven DC migration. We first titrated doses of soluble CD80 on *Cd80^−/−^* BMDCs and compared CD80 expression on cocultured *Pdl1^−/−^* and *Cd80^−/−^* BMDCs to WT BMDCs (fig. S8C). Even in the presence of high amounts of either sCD80 or sPD-1 *Cd80^−/−^*, BMDCs could not promote DC migration in vitro (Fig. 4C).

We previously published and showed (Fig. 3) that when PD-L1 cytoplasmic residues T277, S278, and S279 were converted to alanine, DC migration was significantly impaired both in vitro and in vivo when activated with LPS or polyI:C (*26*). Within these mutated DCs, denoted as *Pdl1^CyMt^*, similar amounts of endogenously expressed PD-L1 and CD80 were up-regulated upon LPS activation compared to WT DCs and the extracellular domain of PD-L1 remained completely intact (fig. S4). Thus, we used this *Pdl1^CyMt^* model to further probe whether administration of exogenous sCD80 or sPD-1 could overcome the PD-L1 intracellular signaling–dependent defect in BMDC migration to CCL21 (Fig. 4D). Within the *Pdl1^CyMt^* BMDCs, neither exogenous sCD80 nor sPD-1 could rescue the defect in migration and no appreciable differences were observed with WT BMDC given the exogenous ligands (Fig. 4D). Collectively, these data suggest that PD-L1 cell-intrinsic signaling within BMDCs promotes migration in a mechanism dependent on cis, not trans, interactions with CD80 and also suggests important features within the intracellular domain that may be important to propagate cellular movement.

### Extracellular and intracellular PD-L1 and CD80 residues are important for chemokine-mediated DC migration

**Fig. 5.**
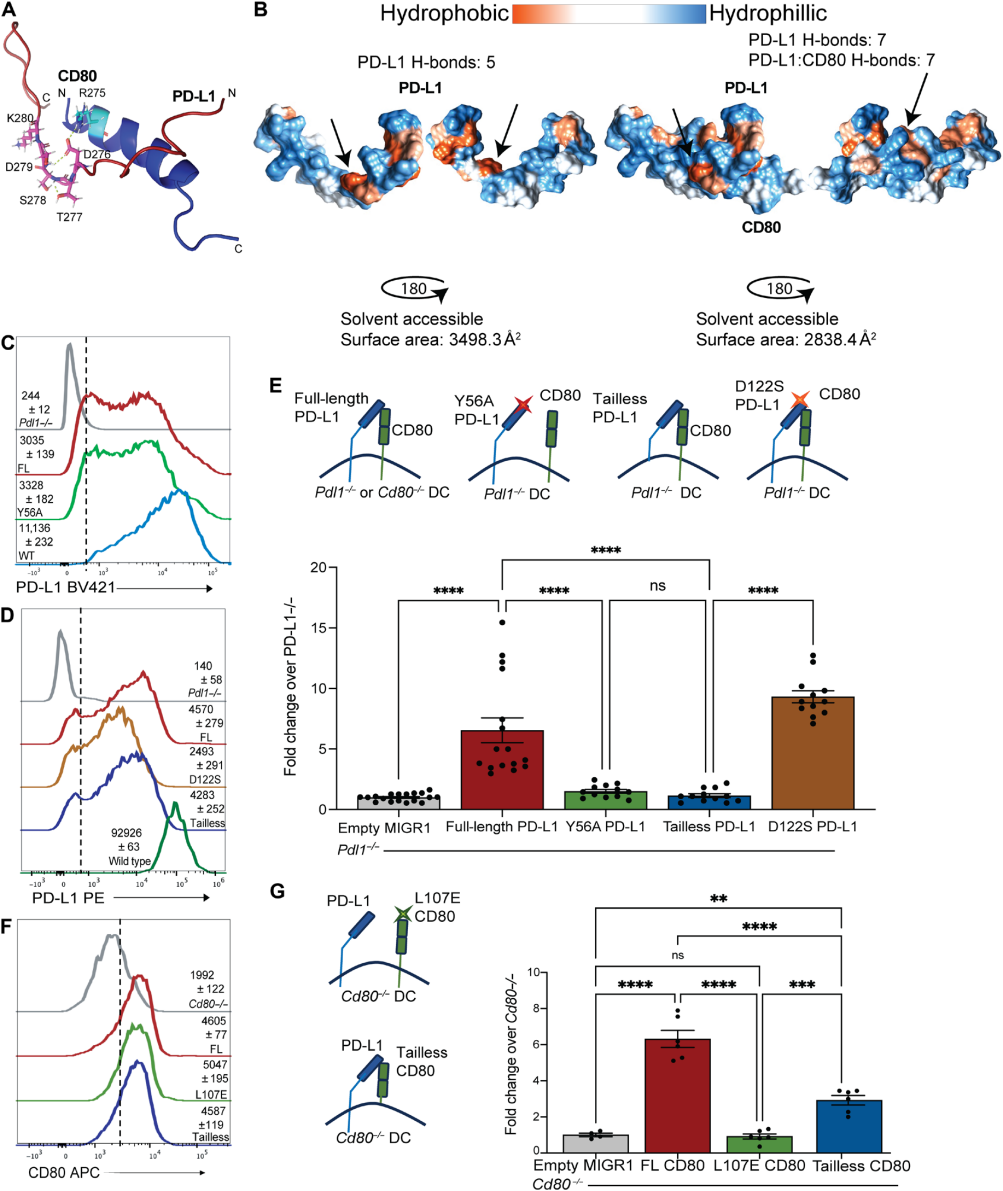
Stabilization of PD-L1:CD80 cis heterodimers through protein-protein interactions facilitates DC migration. (**A**) Computational modeling of between the cytoplasmic tails of murine PD-L1 (residues 265 to 290) and CD80 (residues 270 through 290). Magenta area on PD-L1 represents the DTSSK residues, and gray dotted lines represent hydrogen bonds. (**B**) Surface hydrophobicity modeling of residues shown in (A) of PD-L1 alone or PD-L1 interacting with CD80. Arrows indicate hydrophobic area within TSS curve. (**C** and **D**) Expression of PD-L1 from the MIGR1 MSCV constructs in *Pdl1^−/−^* GM-CSF–derived BMDCs at day 7. PD-L1 antibodies were used to identify PD-L1 expression by flow cytometry. Numbers indicate gMFI on BMDCs with SDs in parentheses. WT PD-L1 td (transduced) is the construct with PD-L1 in the MIGR1 vector, and WT untd (untransduced) is WT PD-L1 BMDCs without the MIGR1 vector. (**E**) Representation of PD-L1 constructs transduced by murine stem cell viral (MSCV) vectors into BMDCs. Fold change of transduced GM-CSF BMDCs (200 ng/ml) with indicated constructs over *Pdl1^−/−^* transduced with empty MIGR1 vector that migrated across a 5-μm transwell to CCL21 (1 μg/ml) after 4 hours. (**F**) Expression of CD80 from the MIGR1 MSCV constructs in *Cd80^−/−^* GM-CSF–derived BMDCs at day 7. Numbers indicate gMFI on BMDCs with SDs in parentheses. WT CD80 td (transduced) is the construct with CD80 in the MIGR1 vector. (**G**) Representation of CD80 constructs transduced by MSCV vectors into BMDCs. Fold change of transduced GM-CSF BMDCs (200 ng/ml) with indicated constructs over *Cd80^−/−^* transduced with empty MIGR1 vector. Data represent two combined experiments with BMDCs derived from at least two mice. Each experiment was completed two or more times with three to six technical replicates per treatment group. Statistical significance was determined using one-way ANOVA with multiple comparisons. **P < 0.01, ***P < 0.001, ****P < 0.0001; ns, *P* > 0.05. Error bars indicate SEM.

The evidence outlined earlier demonstrates a role for cis PD-L1:CD80 heterodimers on activated DCs for directing cellular movement toward a CCL21 chemokine gradient. Both human atezolizumab as well as murine 43H12 and 10F9.G2 αPD-L1 antibodies bind within the extracellular domain of PD-L1 and can disrupt the cis PD-L1:CD80 interaction (*6*). To identify the specific amino acid residues that are necessary for the structural conformation of the PD-L1 and CD80 extracellular and intracellular domains, we evaluated the crystallized structures of PD-L1:CD80 heterodimers and known interacting residue, tyrosine-56 (Y56) on PD-L1 with leucine-107 (L107) on CD80 in mice, which corresponds to Y56 and L106 in human PD-L1 and CD80, respectively (*5*, *32*). Furthermore, the cytoplasmic tail of CD80 has shown its own propensity for cell intrinsic signaling in APCs upon ligation with its binding partners (*27*, *54*, *55*). We modeled the three-dimensional structure of only the cytoplasmic domains of PD-L1 and CD80 to visualize which residues of PD-L1 may interact with CD80 intracellular regions (Fig. 5A). The visualized model is consistent with the structural characterization of the human PD-L1 cytoplasmic domain alone where stability of the monomer relies on electrostatic interactions between the plasma membrane and residues R260, R262, and R265 (*56*). The model predicted several intracellular interactions between the CD80 and PD-L1 cytoplasmic tails, including a hydrogen bond between CD80-R275 and PD-L1^-^ D276 (Fig. 5A). To better visualize how favorable the predicted interaction between the cytoplasmic domains of CD80 and PD-L1 were, we modeled the hydrophobicity of the surface of PD-L1 alone and PD-L1:CD80 bound together (Fig. 5B). We found that the curve in the DTSSK region of PD-L1 appears to facilitate the shape that allows the hydrophobic region to become buried when bound to CD80 (Fig. 5B). The predicted change in total PD-L1 solvent accessible surface area is 3498.2 to 2838.4 Å^2^, demonstrating a likely favorable interaction (Fig. 5B). In addition to the change in solvent accessible surface area, this predicted complex structure results in two additional hydrogen bonds between residues within the PD-L1 molecule (from five to seven) and seven additional hydrogen bonds between PD-L1 and CD80 (Fig. 5, A and B). As polar interactions are considered strong interactions, we questioned whether the defect in DC migration required specific extracellular residues and/or was in part due to the intracellular domain of PD-L1 and CD80.

To begin to address which residues were important for DC migration, we evaluated PD-L1 Y56 (extracellular interaction with CD80), PD-L1 D122S (extracellular interaction with PD-1), and the intracellular tail of PD-L1. We mutated Y56 on murine PD-L1 to alanine and D122 to serine to abolish cis PD-L1 binding with either CD80 or PD-1, respectively, and deleted the cytoplasmic tail of PD-L1 at residue 275 to abolish CD80:PD-L1 intracellular interactions visualized in Fig. 5A (Fig. 5, C to E) (*5*). We used *Pdl1^−/−^* bone marrow to infect stem cells with a retrovirus expressing the mutations outlined above and in Fig. 5E. We observed comparable transduction efficiencies, as assayed by PD-L1 expression in *Pdl1^−/−^* BMDCs, between full-length WT PD-L1, PD-L1 Y56A, PD-L1 D122S, and tailless PD-L1 by anti–PD-L1 staining using gMFI (Fig. 5, C and D). We next assessed chemokine-mediated migration of the transduced BMDCs by using the in vitro transwell assay described above (Fig. 4A). Intriguingly, *Pdl1^−/−^* BMDCs reconstituted with full-length PD-L1 or D122S PD-L1 had elevated levels of migration compared to BMDCs expressing either Y56A PD-L1, or tailless PD-L1 when normalized to *Pdl1^−/−^* BMDCs transduced with an empty murine stem cell virus (MIG) vector (Fig. 5E). In a complementary approach, we used *Cd80^−/−^* BMDCs reconstituted with full-length CD80, L107E CD80, and CD80 with residue 276 replaced with a stop codon (tailless CD80) constructs (Fig. 5, F and G). We found similar CD80 expression across CD80 constructs (Fig. 5F). BMDCs reconstituted with full-length CD80 BMDCs had elevated levels of migration compared to BMDCs expressing L107E, consistent with Y56-L107 extracellular interactions between PD-L1 and CD80 being important for DC migration (Fig. 5G). *Cd80^−/−^* BMDCs reconstituted with tailless CD80 displayed an intermediate migratory phenotype where higher amounts of these BMDCs migrated across a transwell compared to the extracellular binding mutants, but still less than BMDCs expressing full-length CD80 (Fig. 5F). This intermediate phenotype was likely a result of the maintained R275 residue (Fig. 5A); however, this was not directly tested. These findings demonstrate that CD80:PD-L1 extracellular interactions are critical for DC migration. Furthermore, these data predict that the intracellular domains of PD-L1 and CD80 are important for proper CD80:PD-L1 intracellular interactions and downstream signaling events that occur through the cytoplasmic domain of PD-L1. Together, these findings define a clearer picture of the synergistic effects of both intracellular and extracellular interactions between PD-L1:CD80 that are needed to facilitate migration within BMDCs.

### PD-L1 antibody blockade limits DC migration in a B16.F10 tumor model

**Fig. 6.**
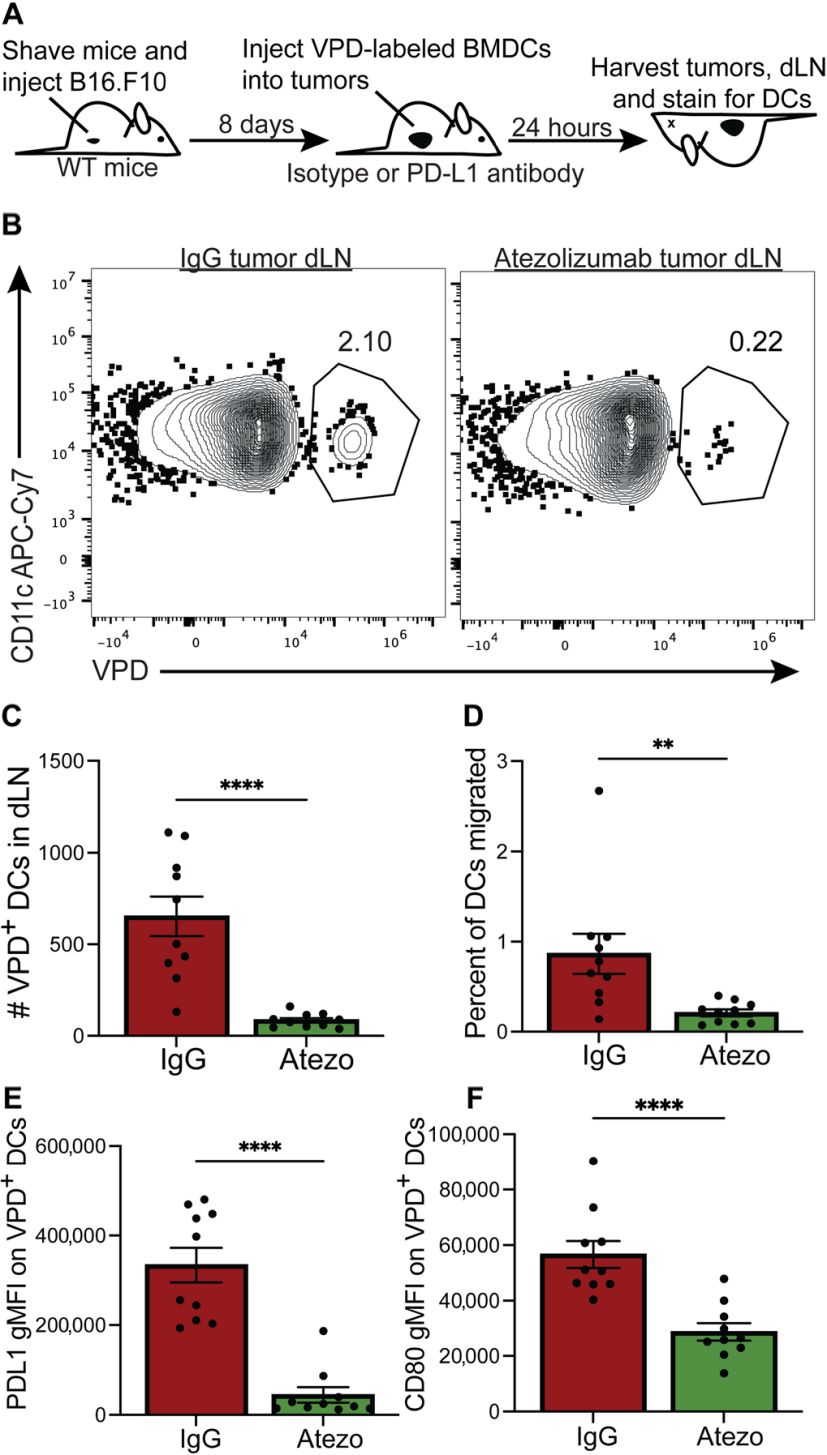
PD-L1 antibody atezolizumab reduces DC migration in a murine melanoma model. (**A**) Experimental scheme. WT mice were injected with atezolizumab antibody or isotype control antibody (200 μg) intraperitoneally 8 days after 2 × 10^5^ B16.F10 cells were injected into the flank intradermally. One day later, the animals were euthanized and VPD-labeled DCs were enumerated. (**B**) Representative flow plots from tumor draining LN in IgG-treated or atezolizumab-treated animals. Shown are live cells. (**C**) Number of transferred VPD-labeled DCs in tumor dLN. (**D**) Percent of DCs migrated was calculated by adding number of VPD-labeled DCs in tumor and dLN. Number of DCs in dLN was calculated as the percent of total DCs in tumor and dLN. (**E**) PD-L1 gMFI of VPD^+^ DCs in dLN. (**F**) CD80 gMFI of VPD^+^ DCs in dLN. The experiment was performed twice, and shown are data from both experiments where *n* = 10 LNs and tumors were evaluated. Each dot represents one LN or LN/tumor pair. Paired Student’s *t* test was performed to calculate the *P* values where **P < 0.01 and ****P < 0.0001. Error bars indicate SEM.

We next determined whether defective DC migration with the human αPD-L1 atezolizumab clone was impeded in a tumor model. αPD-L1 atezolizumab limited human moDC migration (Fig. 1) and interacts with murine PD-L1 in a manner that limits interactions with both PD-1 and CD80 (*57*). Therefore, we asked whether DC migration was impaired in a murine tumor model upon treatment with αPD-L1 atezolizumab. We injected WT mice with B16.F10 melanoma cells intradermally and allowed the tumor to grow for 8 days to approximately 0.5 cm^3^ in size before injecting αPD-L1 atezolizumab or IgG control antibodies and violet proliferation dye (VPD)–labeled BMDCs (Fig. 6A). We transferred approximately 2 × 10^6^ labeled BMDCs intratumorally 24 hours before euthanizing mice (Fig. 6A). To identify transferred BMDCs that migrated from the tumor to the dLN, we enumerated the number of CD11c^+^VPD^+^ BMDCs found in the dLN (Fig. 6, A to C). As in our human moDC migration assays with αPD-L1 atezolizumab (Fig. 1), we found significantly fewer numbers of transferred DCs in the dLN (Fig. 6, B and C). The percent of DCs migrated to the dLN was also significantly reduced in the αPD-L1 atezolizumab–treated mice (Fig. 6D). Furthermore, expression of PD-L1 and CD80 was decreased following αPD-L1 atezolizumab but not IgG control–treated mice (Fig. 6, E and F) similar to murine αPD-L1 antibodies (figs. S2 and S4). Together, these findings indicate that treating mice with αPD-L1 atezolizumab limits DC migration from the tumor to the tumor dLN.

### Selective antibody blockade of PD-L1:CD80 interactions and PD-L1 cytoplasmic mutation ameliorates the severity of inflammatory skin disease in a murine model of psoriasis

**Fig. 7.**
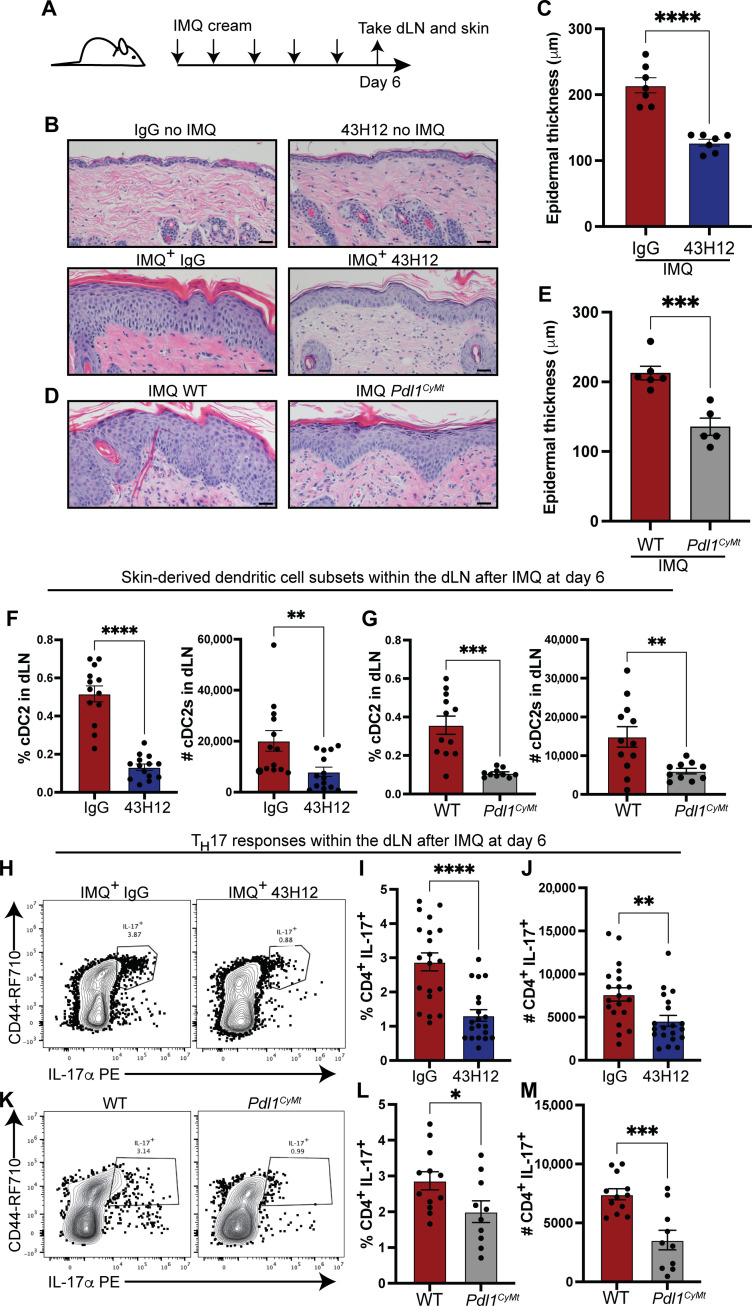
Dissociation of PD-L1:CD80 cis interactions ameliorates the severity of chronic skin inflammation. (**A**) Experimental scheme for Fig. 6 (B to M). (**B**) Representative hematoxylin and eosin (H&E) images from healthy and lesional skin of IgG and 43H12 antibody–treated mice. (**C**) Quantification of epidermal thickness of lesional skin from IgG and 43H12 antibody–treated mice in micrometer. (**D**) As in (B), except WT and *Pdl1^CyMt^* mice. (**E**) As in (C) except WT and *Pdl1^CyMt^* mice. (**F** and **G**) Percentage and numbers of CD11b^+^ cDC2s in skin-dLNs from IMQ-treated mice at day 6 from indicated groups. (**H**) Representative flow plots showing decreased percentages of IL-17^+^ cells in the skin-dLNs of 43H12 antibody–treated mice 6 days after IMQ treatment. (**I**) Percentage and (**J**) numbers of IL-17^+^ CD4^+^ T cells in skin-dLNs from IMQ-treated mice at day 6. (**K**) As in (H) except WT and *Pdl1^CyMt^* mice. (**L**) Percentage and (**M**) numbers of IL-17^+^ CD4^+^ T cells in WT and *Pdl1^CyMt^* mice 6 days after IMQ treatment. Scale bars, 100 μm in (B) and (D). Data represent two to three combined experiments performed on separate occasions with two to four mice per group. Each dot represents skin from one mouse (C and E) or cells from one dLN (D to M). Statistical significance was determined using Student’s paired *t* test. *P < 0.05, **P < 0.01, ***P < 0.001, ****P < 0.0001; ns, *P* > 0.05. Error bars indicate SEM.

We next asked whether, in a model of psoriasis where excessive inflammation is detrimental to disease outcomes, whether we could prohibit DC migration and dampen inflammatory responses. We used the murine model of psoriasiform-like dermatitis induced by topical application of imiquimod (IMQ), a TLR7 agonist (Fig. 7A and fig. S9A) (*58*). We confirmed within the KikGR model that after two consecutive days of topical IMQ application and intraperitoneal PD-L1 43H12 antibody injection that less KikRed^+^ DCs migrated to the skin-dLNs by both number and frequency (fig. S9B) and that there was less surface-associated staining of PD-L1 and CD80 on the KikRed^+^ DCs in the dLN after 2 days of topical IMQ application compared to the IgG isotype control mice (fig. S9C). After five consecutive days of topical IMQ application, we observed increased epidermal thickening, a result of keratinocyte hyperproliferation, compared to untreated mice (Fig. 7, B to E). In contrast, treatment with IMQ, at the same route and dose of PD-L1 43H12 antibody that decreased DC migration (Fig. 3), resulted in significantly less epidermal thickening after IMQ treatment compared to isotype control–treated mice (Fig. 7, B and C). These changes were only observed in IMQ-treated skin (Fig. 7B). We also observed decreased epidermal thickening after IMQ treatment in *Pdl1^CyMt^* mice compared to WT control mice although to a slightly lesser degree than 43H12-treated mice (Fig. 7, B to E).

To validate in this model that there was a disruption in DC localization after 5 days of topical IMQ treatment within αPD-L1 43H12–treated mice and *Pdl1^CyMt^* mice, we evaluated DC subset frequency and number in the dLN. Following IMQ, fewer cDC2s were observed in the dLN in PD-L1 43H12 antibody–treated mice, as compared to IgG control mice (Fig. 7F) and *Pdl1^CyMt^* mice (Fig. 7G). There were reduced amounts of surface-associated PD-L1 and CD80 on cDC2s in αPD-L1 43H12–treated mice but comparable amounts of PD-L1 and CD80 on cDC2s *Pdl1^CyMt^* mice (fig. S9). There were also similar amounts of cDC1s that expressed slightly higher amounts of CD80 within αPD-L1 43H12 mice compared to IgG mice after IMQ treatment but fewer cDC1s in *Pdl1^CyMt^* mice (fig. S9). We also observed less surface-associated PD-L1 on DCs after PD-L1 43H12 antibody treatment, likely due to the steric hindrance of these antibodies (fig. S9). We did observe differences in CD80 expression on cDC1s within the dLN when comparing αPD-L1 43H12 and IgG control mice (fig. S9). In *Pdl1^CyMt^* mice, we observed similar amounts of PD-L1 and CD80 on cDC1s (fig. S9). We also observed fewer Langerhans cells (LCs) (fig. S9) in 43H12 antibody–treated mice compared to IgG control mice, yet comparable amounts of LCs in *Pdl1^CyMt^* mice (fig. S9). The LCs within the dLN of αPD-L1 43H12–treated mice had reduced surface-associated PD-L1 (fig. S9) and comparable levels of CD80 (fig. S9), but equal amounts on LCs in *Pdl1^CyMt^* mice (fig. S9).

Last, we enumerated the frequency and number of IL-17–producing CD4^+^ T cells within the dLN and observed a reduction in frequency and number of IL-17–producing CD4^+^ T cells after ex vivo stimulation of cells from the dLN of αPD-L1 43H12–treated mice (Fig. 7, H to J) and *Pdl1^CyMt^* mice (Fig. 7, K to M). Together, these findings reveal that blocking cis PD-L1:CD80 interactions through PD-L1 antibodies can limit DC migration to the dLN, decrease the amount of IL-17–producing CD4^+^ T cells, and decrease the extent of chronic inflammatory skin disease. Furthermore, these findings highlight the importance of PD-L1 intrinsic signaling to enforce DC migration, epidermal thickening, and IL-17 production by CD4^+^ T cells.

## DISCUSSION

The study builds on our previous work defining the importance of the intracellular domain of PD-L1 in regulating chemokine-mediated dermal DC migration (*26*). In this study, we expand on these findings by demonstrating that like PD-L1, CD80 was required for efficient DC migration to the dLN and that blocking PD-L1:CD80 cis interactions could lessen the severity of IMQ-induced psoriasis driven by dermal DCs (*31*). As CD80 interacts in cis with PD-L1 during DC activation, we hypothesized that interactions between PD-L1 and CD80 were required for optimal DC migration necessary for the initiation of downstream T cell responses. We identify that the regulation of DC migration by PD-L1 intracellular signals requires interactions with CD80 in cis. We found that both the extracellular and intracellular domains of CD80 were necessary for interactions with PD-L1 and that mutation of either of these domains attenuated migration of DCs in vitro. In a system where ligands of PD-L1 could be added exogenously, we found that neither soluble PD-1 nor CD80 could rescue migration, consistent with our hypothesis that both the intracellular and extracellular domains of CD80, but not PD-1, were necessary. We also found that following either 10F9.G2 or 43H12 PD-L1 antibody injection, skin-derived DCs had limited migration to the cutaneous LNs and expressed significantly lower levels of CD80. Furthermore, we document how the αPD-L1 antibody atezolizumab prevents DC migration of intratumorally transferred DCs. The findings point to a dual action of αPD-L1 antibodies, which consists of blocking both PD-L1:PD-1 interactions in trans between T cells and DCs and disrupting PD-L1:CD80 cis interactions, thus freeing CD80 to interact with CTLA-4 (*6*, *10*, *43*, *59*). Therefore, the notion that high expression of both PD-L1 and CD80 could drive activated DCs to the dLN to prime naïve T cells further emphasizes how similar proportions of PD-L1 and CD80 can result in stimulation rather than inhibition of adaptive immunity.

Migratory DCs are particularly powerful regulators of T cell activation due to the high amounts of MHC and costimulatory molecules expressed (*16*). Therefore, the skewing of DC trafficking capacities from peripheral tissues to dLNs where T cells reside remains a critical determinant of initiation of adaptive immune responses (*22*, *60*). Ovcinnikovs *et al.* demonstrated that migratory DCs are the primary target for modification of CD80 levels by CTLA-4 on regulatory T cells (T_regs_) at steady state, and, thus, it is conceivable that an additional regulatory mechanism exists by which tissue-resident T_regs_ continuously modify and limit PD-L1:CD80 interactions on DCs to restrain excess amounts of migration and subsequent antigen presentation in the absence of an inflammatory stimulus (*61*, *62*). Although not evaluated in this study, the effects of blocking CD80 trans-endocytosis with αCTLA-4 antibodies could also affect DC migration. On the basis of our studies, we would predict that blocking CTLA-4 may promote intracellular CD80 and PD-L1 signals that may increase DC migration, and it is possible that in the clinic, this is a mechanism that affects the efficacy of CTLA-4 blockade in a tumor setting. Given that mobilization of activated DCs to dLNs is a key feature in tissue-specific immunity, further investigation of how PD-L1 and CD80 influences DC trafficking within other inflammatory contexts and other organ systems should be assessed. Thus far, we have only tested DC egress from the skin in the context of a local inflammatory stimulus including polyI:C injection, IMQ treatment, and a melanoma model. Yet, evidence exists for the ubiquitous role that chemokine signaling plays in DC migration across several tissues such as the liver, intestines, lung, and kidneys for immune surveillance and tissue homeostasis (*22*, *63*, *64*).

The pathogenesis of cutaneous immune-related adverse events (irAEs) observed in patients with cancer treated with immune checkpoint inhibitors remains variable, with one critical determinant being whether patients are treated with PD-1, PD-L1, and CTLA-4 antibodies (*65*, *66*). Up to 40% of patients with cancer treated with immune checkpoint inhibitors develop skin-related irAEs, including psoriasiform-like dermatitis marked by large amounts of infiltrating lymphocytes (*67*–*70*). However, there are no studies that have directly evaluated specific differences between PD-1 and PD-L1 antibodies in psoriasiform-like dermatitis associated with checkpoint inhibitors. Here, we document that interruption of PD-L1:CD80 interactions and PD-L1 intrinsic signals, leaving PD-L1:PD-1 interactions intact, leads to a less inflammatory phenotype within the context of psoriasiform-like skin disease in mice, supporting the idea that loss of PD-L1 regulated DC migration improves psoriasiform outcomes when PD-1–binding remains intact. Sugiura *et al.* (*5*, *11*) demonstrate that direct blockade of cis PD-L1:CD80 alleviated autoimmune processes across several different tissue types due to decreased T cell activation and infiltration into inflamed tissue. Antibodies that block only PD-L1:CD80 cis interactions are intended to bolster PD-L1:PD-1 signaling while limiting activation of T cells. This limited activation of T cells happens as a result of blocking PD-L1:CD80 heterodimers that bind in a complex with CD28 as previously documented (*5*). However, DC migration was not assessed in these models; therefore; it remains a possibility that an additional mechanism for reduced skin autoimmune disease is via limited DC migration and therefore limited availability of self-antigen presentation by migratory DCs in the dLN.

Our data highlight the need to evaluate differences in patients treated with PD-1 and PD-L1 antibodies regarding irAEs since signaling of PD-L1 within DCs promotes migration from the tissue to the draining LN. These previously unidentified findings further elaborate the emphasis of cis PD-L1:CD80 interactions on migratory DCs as a primary target for PD-L1 antibodies across several models of skin inflammation, which leads to a significant decrease of CD80, unlike PD-1 antibody therapy (*6*, *71*, *72*). However, simultaneous treatment of mice with PD-L1 and CTLA-4 antibodies allows for restored expression of CD80 on tumor-associated DCs, leading to increased CD28 costimulation, downstream T cell responses, and reduced overall tumor burden (*6*, *72*, *73*). As suggested above, this could be a consequence of promoting CD80 surface levels and increasing DC migration. Although conflicting evidence exists for whether cis PD-L1:CD80 heterodimers or CD80 alone is more efficient at inducing T cell responses (*71*, *74*), our data suggest that in tumors with a high DC signature, PD-L1 therapies may be more beneficial as it may prolong neoantigen-bearing DC localization in the tumor. Addition of CTLA-4 antibodies could also act to preserve high amounts of CD80 to reinvigorate exhausted T cells (*75*). Alternatively, if PD-L1 monotherapy is to be used within the context of cancer, rational design of new PD-L1 antibodies should consider binding properties that exclusively block PD-1–binding domain while sparing the CD80-binding domain.

Furthermore, dissociating the cis PD-L1:CD80 heterodimers with the intention of retaining DCs in peripheral tissues is a promising therapeutic avenue in disease contexts where DC-driven downstream adaptive immune responses are unfavorable, such as in the context of transplantation and autoimmune disease, like psoriasis (*76*, *77*). This study serves as a rational for why specifically targeting PD-L1:CD80 cis interactions may work and further pursuing these costimulatory and coinhibitory molecules in the development of new interventions could lead to progress in the repurposing of current immune checkpoint therapies across a range of immunologic diseases.

## MATERIALS AND METHODS

### Sex as a biological variable

In studies involving human blood samples and mice, males and females were randomly assigned to each treatment group. We have not observed significant differences between sexes throughout our studies.

### Statistical analysis

Unpaired Student’s *t* tests and one-way analysis of variance (ANOVA) were used. Software Graphpad Prism 10 was used for statistical analysis. All error bars displayed are the SEM. Please see figure legends for details.

### Study approval

All animal procedures were approved by the Institutional Animal Care and Use Committee at the University of Colorado Anschutz Medical Campus under study approval number 67. All patients provided written and informed consent, and the study was approved by the institutional review board at the University of Colorado-Anschutz under COMIRB # 17-2159 and 06-0566.

### Data availability

This study did not generate any unique datasets or code.

### Mice

Six- to eight-week-old C57BL/6 “WT” mice were bred in-house or purchased from Charles River NCI. *Cd80^−/−^* mice were rederived from frozen embryos at the Jackson Laboratory and were further bred in-house. *B7^−/−^*, KikGR, *Pdl1^−/−^*, and *Pdl1^CyMt^* mice were all bred in-house at the University of Colorado Anschutz Medical Campus Animal Facility. All strains of mice used in this study were bred on a C57BL/6 background. Male and female mice were used in these studies with no observed gender differences. All mice were group housed in a room with stable temperatures and humidity, with a 12-hour light cycle. Mice had free access to food and water. All animal procedures were approved by the Institutional Animal Care and Use Committee at the University of Colorado.

### Human blood samples

Deidentified peripheral blood samples from healthy donors were obtained from the Human Immune Tissue Network Biobank (COMIRB # 17-2159) and were collected at the University of Colorado Clinical and Translation Research Center, which is a part of the Colorado Clinical and Translation Sciences Institute. Other samples were selected from a biorepository of patients who had undergone liver transplantation and collected under the IRB protocol 06-0566 and protocol 23-2301 (table S1). All patients provided written and informed consent and the study was approved by the institutional review board at the University of Colorado-Anschutz.

### Generation of human monocyte–derived BMDCs

Whole blood from healthy donors was collected, and PBMCs were isolated using Lymphoprep according to the manufacturer’s instructions. Cells were resuspended in 2.5% fetal bovine serum (FBS) (R&D systems) in 1× phospho-buffered saline (PBS) (Corning) and counted before further isolation. CD14^+^ PBMCs were isolated using a magnetic cell sorting separation protocol using liquid chromatography columns, anti–phycoerythrin (PE) microbeads (Miltenyi Biotec), and PE-conjugated CD14^+^ antibody (BioLegend, clone HCD14) according to the manufacturer’s instructions. Freshly isolated CD14^+^ cells were stimulated with recombinant human IL-4 (250 U/ml, SinoBiological) and GM-CSF SinoBiological (100 U/ml) in RPMI 1640 containing 10% FBS and 1:100 each of Hepes (Corning), sodium pyruvate (Corning), nonessential amino acids (NEAA, Corning), 2-mercaptoethanol (Fisher Chemical), and penicillin-streptomycin (Corning) at days 0, 2, and 4, and, at day 5, moDCs were collected and used for downstream applications. For T cell stimulation, the cells were treated with phorbol 12-myristate 13-acetate and ionomycin (BioLegend) at 50 ng/ml for 6 hours before flow cytometric analysis.

### Protein structures and PyMOL modeling

Human protein structures with PD-L1 bound to PD-1 (PBD ID: 4ZQK) and human PD-L1 bound to CD80 (PBD ID: 7TPS) were analyzed for interactions using PyMOL software developed by Schrödinger Inc. In addition, the PD-L1 IgV extracellular domain (PBD ID: 5C3T), atezolizumab (PBD ID: 5X8L), avelumab (PDB ID: 5GRJ), and durvalumab (PBD ID: 5X8M) structures were all modeled with readily available crystal structures on PDB using PyMOL. Mouse PD-L1 and CD80 tail structures were predicted using Phyr2 Protein Fold Recognition. Potential binding interface was predicted using RosettaDock 4.0 available at https://rosettacommons.org/ for backbone protein-protein interactions (*78*). Hydrogen bonds were identified in PyMol, the hydrophobicity surface coloring was identified, and images were generated using UCSF Chimera software.

### FITC paint assay

Mice were anesthetized and shaved in two spots on the upper dorsum. Twenty-four hours later, the mice were anesthetized and topical application of 20 μl of FITC paint per shaved site was used. FITC paint consisted of FITC (5 mg/ml, Sigma-Aldrich) emulsified in a 1:1 ratio solution of dibutyl phthalate (Sigma-Aldrich) and acetone.

### KikGR photoconversion

KikGR mice were anesthetized and shaved in two spots on the upper dorsum. Twenty-four hours later, the mice were anesthetized and photoconverted through exposure to violet light for 1 min per shaved site using a 405-nm 100 mW/cm^2^ handheld laser pointer.

### PolyI:C injection and in vivo use of antibodies in mice

PolyI:C (Invivogen) was injected intradermally into the dorsal skin bilaterally of mice at a concentration of 5 μg/ml in PBS at a final volume of 50 μl per injection site (*26*). For systemic administration of antibodies, anti–PD-1 (Selleckchem clones RMP1-14 and 29F.1A12), IgG isotype control (clone LTF-2), and PD-L1 antibodies including the 43H12 clone (a gift from Y. Zhu) and 10F9.G2 (BioXCell) were injected intraperitoneally in a final volume of 200 μl of PBS at a concentration of 200 μg/ml.

### LN harvesting

Murine LNs were harvested, minced with needles, and digested with collagenase D (Worthington Biochemical) (1 mg/ml) and deoxyribonuclease I (Thermo Fisher Scientific) (0.25 mg/ml) in Click’s medium (Fuji Film) for 30 min at 37°C (*26*). The cells were passed through a 100-μm nylon mesh cell strainer (FisherBrand) and washed with 5 mM EDTA (Invitrogen) in Click’s medium containing 2.5% FBS to stop the digestion. The cells were pelleted and washed before staining for flow cytometry.

### BMDC cultures

Whole bone marrow from femurs and tibias of mice were isolated and RBC lysed. Cells were plated in minimum essential medium (MEM, Corning) containing 10% FBS and Hepes, sodium pyruvate, 2-mercaptoethanol, NEAA, and penicillin-streptomycin at a concentration of 1 × 10^6^ cells/ml and cultured in GM-CSF (20 ng/ml) for 7 days. Medium containing GM-CSF (20 ng/ml) was changed every 2 days, and the cells were harvested at day 7 by dissociating loosely adherent cells through agitation by pipetting. For Flt3L-matured BMDCs, bone marrow cells were plated at a concentration of 1 × 10^6^ cells/ml in a 10-cm dish, cultured with Flt3L (50 ng/ml, Peprotech), and topped off with 10 ml of Flt3L (100 ng/ml) at day 3. The cells were harvested by gently pipetting at day 8 for downstream applications.

### Transwell assays

Human moDCs and murine BMDCs were treated with LPS (Invivogen) at a concentration of 200 ng/ml) for 4 hours. Murine or human CCL21 (Peprotech) were placed in the bottom well at a concentration of 1 μg/ml in a total volume of 500 μl of complete MEM (mouse) or RPMI 1640 (human). Human moDCs (5 × 10^4^ cells in complete RPMI 1640) and murine BMDCs (1 × 10^5^ in complete MEM) were placed in the top transwell. For human experiments, 8-μm pore transwells (Corning) were used, and for mouse experiments, 5-μm pore transwells (Corning) were used. After 4 hours, the medium from the bottom well was removed and spun, and the cells were counted using a hemocytometer.

### Cloning and plasmids

The *Pdl1* (*Cd274*) gene was amplified from a pBABE plasmid construct containing *Pdl1* cDNA with designed primers to introduce restriction sites for Ale1 at the 5′ end and HindIII at the 3′ end using polymerase chain reaction. The *Cd80* gene was amplified from a mouse CD80/B7-1 gene ORF cDNA clone in a pGEM-T plasmid vector (Sino Biological) using primers containing restriction sites at the 5′ end with Xho1 sequence and EcoRI sequence for the full-length CD80 primers. Tailless CD80 primers included the EcoRI restriction site at the 5′ end and the Not1 restriction site at the 3′ end. Restriction digestions were performed, and agarose gels were run for verification of the correct digestions. Ligation of the purified DNA were performed to insert either *Pdl1* or *Cd80* cDNA into the MIGR1 murine stem cell viral (MSCV) plasmid vector using the Rapid DNA ligation kit according to manufacturer’s instructions as well as transformation of competent bacteria (Roche). Colonies grown were selected for minipreps (Qiagen) and sent for sequencing at the Barbara Davis Bioresource/Molecular Core using Gag sequencing primers. Point mutations for Y56A PD-L1, PD-L1 D122S, and L107E CD80 were introduced into PD-L1 and CD80 cDNA within the MIGR1 plasmids by using mutagenic primers (IDT) and Q5 Site-Directed Mutagenesis Kit (Agilent). Tailless constructs were made via standard cloning using primers to replace residue 276 with a stop codon so that the CD80 extracellular domain and transmembrane domain remained intact, but the C terminal tail was absent after residue 275. Similarly, tailless constructs for PD-L1 were made via standard cloning using primers to replace residue 275 with a stop codon. Transformation of competent bacteria (New England Biolabs) and minipreps (Qiagen) were prepared for mutant plasmid sequencing. Maxipreps (Qiagen) to obtain large quantities of full-length PD-L1, Y56A PD-L1, full-length CD80, L107E CD80, and tailless CD80 MSCV vectors were used.

### Cell lines and transfection

PlatE cells were maintained in Dulbecco’s modified Eagle’s medium (DMEM, Corning) and supplemented with 10% FBS, penicillin-streptomycin, sodium pyruvate, Hepes, NEAA, and 2-mercaptoethanol. Further antibiotics used for PlatE selection were blasticidin at 10 μg/ml (Invivogen) and puromycin at 1 μg/ml (Invivogen). Cells (2 × 10^6^) were plated in 10 ml of media in a 10-cm dish overnight before transfection with retroviral gene vectors. PlatE cells were transfected using 6 ml of transfection media (DMEM with Hepes, NaPyr, NEAA, and 2-mercaptoethanol) and 3 ml of Opti-MEM (Thermo Fisher Scientific) containing 20 μl of Lipofectamine 2000 (Thermo Fisher Scientific) and 10 μg of MSCV DNA per 10-cm plate and incubated for 6 hours. One milliliter of FBS was added and incubated overnight. Medium was replaced with complete DMEM virus collection media (Hepes, NEAA, BME, and FBS), and 48 hours later, viral supernatant was collected.

### Retroviral gene transduction

GM-CSF BMDCs were collected at day 4, spun down, and counted, and 2.5 × 10^6^ cells were spinfected for 2 hours at room temperature in 1 ml of collected viral supernatant containing polybrene at a concentration of 15 μg/ml (Sigma-Aldrich). Pelleted cells were resuspended in GM-CSF containing media, plated in 10-cm plates, and collected at day 7 for downstream applications.

### B16.F10 melanoma cell line and BMDC transfer

B16.F10 melanoma cells were cultured in DMEM (Corning) and supplemented with 10% FBS, penicillin-streptomycin, sodium pyruvate, Hepes, NEAA, and 2-mercaptoethanol. Before injection, the cells were washed, counted, and injected at 2 × 10^6^ total cells per injection site dissolved in Matrigel (Corning). At day 8 after injection, GM-CSF-derived BMDCs were labeled with violet proliferation dye (BD Biosciences) and 2 × 10^6^ cells were injected per melanoma. Twenty-four hours later, tumors and tumor dLNs were harvested. Tumors were digestion in Click’s media (Fuji Film) containing hyaluronidase (0.35 mg/ml, Sigma-Aldrich) and collagenase IV (1 mg/ml, Sigma-Aldrich) for 1 hour at 37°C. Digestion was stopped with Click’s medium containing 5 mM EDTA and 2.5% FBS, and the cells were washed and used for downstream flow cytometric analysis.

### Topical IMQ application

For IMQ studies, 6- to 8-week mice (both male and female) were anesthetized with isoflurane before shaving of back skin and IMQ treatment. Twenty-five milligrams of IMQ cream (5%, Perrigo) was applied to shaved dorsal skin, and application was repeated for five total days as previously described (*58*). Measurements of epidermal thickness were performed in Adobe Photoshop. Using a ruler acquired at the same magnification and the measurement tool in Adobe Photoshop, the epidermis was measured. At least six images were taken from each sample, and four to six measurements from each image were taken to provide an average epidermal thickness measurement per sample. Each dot represents this average epidermal thickness per animal.

### Flow cytometric analysis

Cells (BMDCs or single-cell LN suspensions) were stained for flow cytometry with antibodies diluted in mouse Fc block (24G2) or human Fc block (Miltenyi Biotech) for 30 min at 4°C. Following staining, the cells were washed and run on the cytometer. Unless otherwise noted, all flow cytometry antibodies were purchased from BioLegend. For murine DCs, BMDCs and T cells, the cells were stained with CD45 (clone 30-F11), B220 (clone RA3-6B2), CD11c (clone N418), MHC class II (clone M5/114.15.2), XCR1 (clone ZET), CD11b (M1/70), PD-L1 (clone 10F9.G2), CD80 (clone 16-10A1), CD86 (clone GL-1), CCR7 (clone 4B12), CD4 (clone RM4-5), IL-17a (clone TC11-18H10.1), and anti–phospho-ERK1/2 (clone D13.14.4E). For human moDC staining, the cells were stained using CD14 (clone HC14), CD209 (clone 9E9A8), PD-1 (clone EH12.2H7), PD-L1 (clone 29E.2A3), CD80 (clone W17149D), CCR7 (clone G043H7), CD86 (clone IT2.2). For F-actin staining of murine BMDCs, the F-actin Visualization Biochem Kit (fluorescence format) from Cytoskeleton Inc. was used. BMDCs were fixed, permeabilized, and stained with Phalloidin (PE from kit or AF647, Invitrogen). All samples were collected on a Beckman Coulter CytoFLEX LX flow cytometer with CytExpert software and analyzed with FlowJo software (Treestar).
