# Supplementary material for: PD-L1-CD80 interactions are required for intracellular signaling necessary for dendritic cell migration
Source: Sci Adv. 2025 Jan 29;11(5):eadt3044. doi: 10.1126/sciadv.adt3044 (PMC11777207; doi:10.1126/sciadv.adt3044)
Supplement: Supplementary file 1 — Figs. S1 to S9 Table S1 Legend for data S1 [file sciadv.adt3044_sm.pdf]

Science Advances
AAAS

Supplementary Materials for

PD-L1-CD80 interactions are required for intracellular signaling necessary for dendritic cell migration

Uma Kantheti *et al.*

Corresponding author: Beth Ann Jirón Tamburini, beth.tamburini@cuanschutz.edu

Sci. Adv. **11**, eadt3044 (2025)
DOI: 10.1126/sciadv.adt3044

The PDF file includes:

Figs. S1 to S9
Table S1
Legend for data S1

Other Supplementary Material for this manuscript includes the following:

Data S1

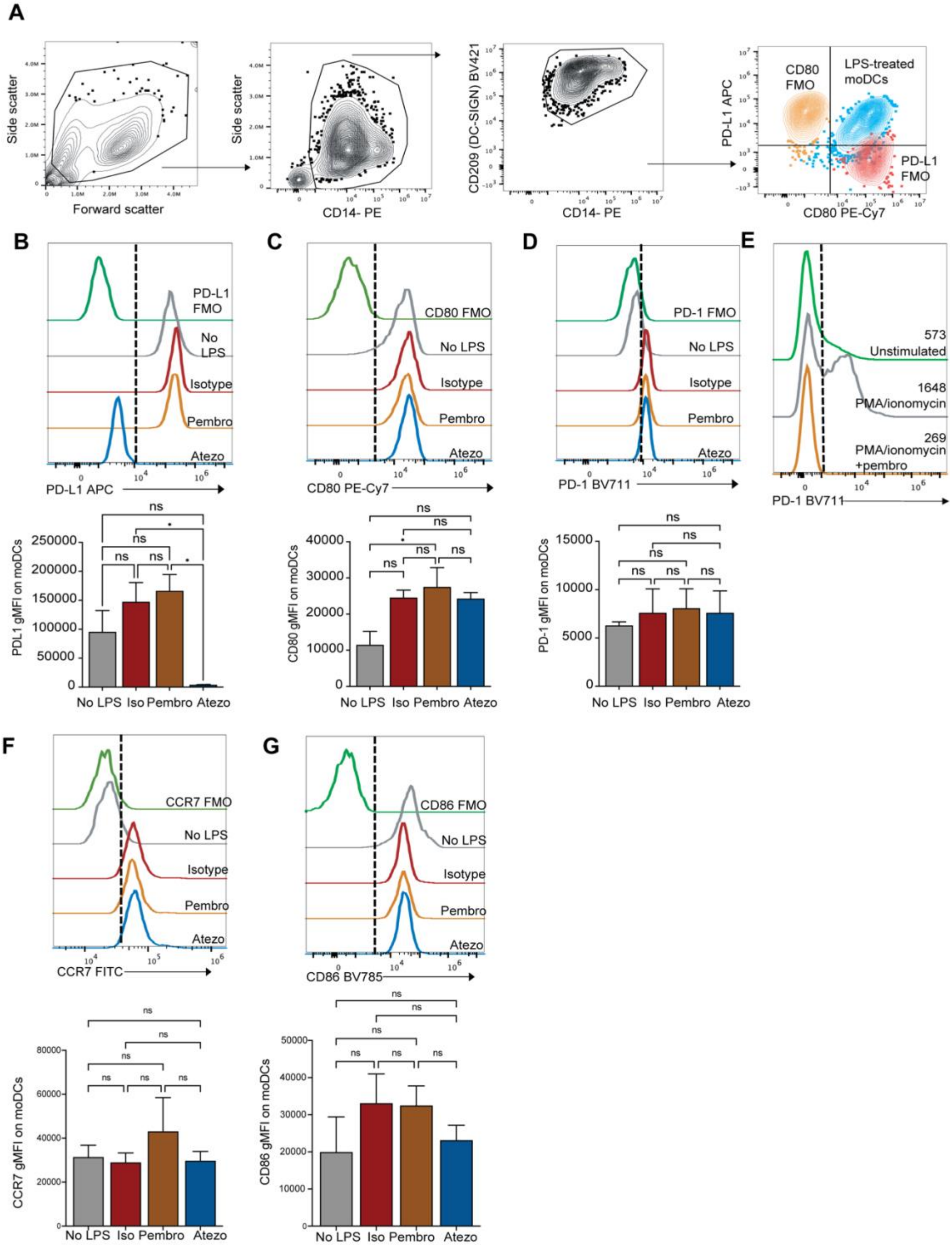

S1. Monocyte-derived DCs treated with humanized IgG antibodies exhibit no difference in CD80, PD-1, CCR7 or CD86 surface expression.

A) Gating strategy for moDCs differentiated from human blood 5 days after isolation. The rightmost plot show gating for PD-L1 and CD80 using fluorescence minus one (FMO).

B-D) PD-L1, CD80 and PD-1 surface expression on moDCs after 4 hours LPS stimulation. The experiment was repeated two times with similar results.

E) Anti-human CCR7 receptor staining indicating presence surface-associated CCR7 after 4 hours of treatment on LPS-stimulated moDCs.

F) CD86 surface expression on LPS-treated moDCs with untreated moDCs, and LPS-treated IgG isotype, pembrolizumab and atezolizumab.

Data shown in figure is representative of 3 independent experiments with 2-4 replicates per treatment group.

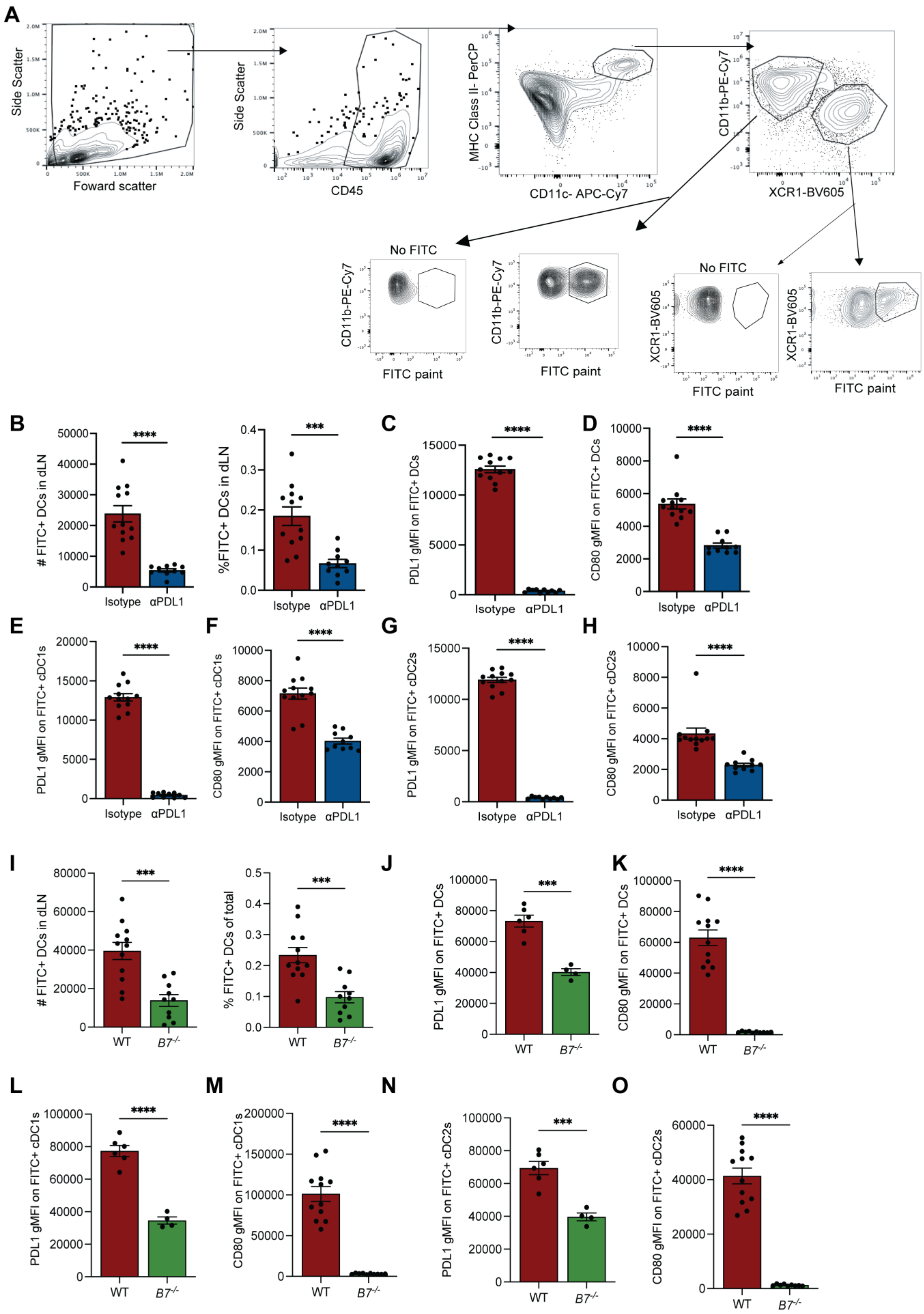

A
Side Scatter
Foward scatter
Side Scatter
CD45
MHC Class II- PerCP
CD11c- APC-Cy7
CD11b-PE-Cy7
XCR1-BV605
No FITC
CD11b-PE-Cy7
FITC paint
CD11b-PE-Cy7
FITC paint
No FITC
XCR1-BV605
FITC paint
XCR1-BV605
FITC paint
B
FITC+ DCs in dLN
Isotype αPDL1
%FITC+ DCs in dLN
Isotype αPDL1
C
PDL1 gMFI on FITC+ DCs
Isotype αPDL1
D
CD80 gMFI on FITC+ DCs
Isotype αPDL1
E
PDL1 gMFI on FITC+ cDC1s
Isotype αPDL1
F
CD80 gMFI on FITC+ cDC1s
Isotype αPDL1
G
PDL1 gMFI on FITC+ cDC2s
Isotype αPDL1
H
CD80 gMFI on FITC+ cDC2s
Isotype αPDL1
I
FITC+ DCs in dLN
WT B7-/-
% FITC+ DCs of total
WT B7-/-
J
PDL1 gMFI on FITC+ DCs
WT B7-/-
K
CD80 gMFI on FITC+ DCs
WT B7-/-
L
PDL1 gMFI on FITC+ cDC1s
WT B7-/-
M
CD80 gMFI on FITC+ cDC1s
WT B7-/-
N
PDL1 gMFI on FITC+ cDC2s
WT B7-/-
O
CD80 gMFI on FITC+ cDC2s
WT B7-/-

S2. PD-L1 antibody-treated mice have fewer FITC+ DCs and less PD-L1 and CD80 surface expression in the dLN 24 hours after polyI:C injection.

A) Gating strategy for dermal-derived migratory DC subsets in mice after polyI:C injection.

B) Number and percentage of FITC+ DCs (combined cDC1s and cDC2s) in the dLN 24 hours after intradermal polyI:C injection and topical FITC paint application.

C & D) Surface expression of PD-L1 and CD80 evaluated by geometric mean fluorescence intensity (gMFI) on FITC+ DCs in the dLN 24 hours after intradermal polyI:C injection and topical FITC paint application

E & F) Surface expression of PD-L1 and CD80 evaluated by gMFI on FITC+ cDC1s in the dLN 24 hours after intradermal polyI:C injection and topical FITC paint application

G & H) Surface expression of PD-L1 and CD80 evaluated by gMFI on FITC+ cDC2s in the dLN 24 hours after intradermal polyI:C injection and topical FITC paint application

I) Number and percentage of FITC+ DCs (combined cDC1s and cDC2s) in the dLN 24 hours after intradermal polyI:C injection and topical FITC paint application.

J & K) Surface expression of PD-L1 and CD80 evaluated by geometric mean fluorescence intensity (gMFI) on FITC+ DCs in the dLN 24 hours after intradermal polyI:C injection and topical FITC paint application

L & M) Surface expression of PD-L1 and CD80 evaluated by gMFI on FITC+ cDC1s in the dLN 24 hours after intradermal polyI:C injection and topical FITC paint application

N & O) Surface expression of PD-L1 and CD80 evaluated by gMFI on FITC+ cDC2s in the dLN 24 hours after intradermal polyI:C injection and topical FITC paint application

Data shown in figure represents 2 combined experiments with 4-6 LNs per group. Each dot represents one lymph node where 2-3 mice per group were used. For L and N PD-L1 MFI is only shown from one experiment due to the use of a different fluorophore in the repeat experiment being of different intensity. Experimental repeat showed similar differences as shown in figure. Statistical significance was determined using student’s t-test. *$p<0.05$, **$p<0.01$, ****$p<0.0001$; n.s., $p>0.05$. Error bars indicate standard error of the mean.

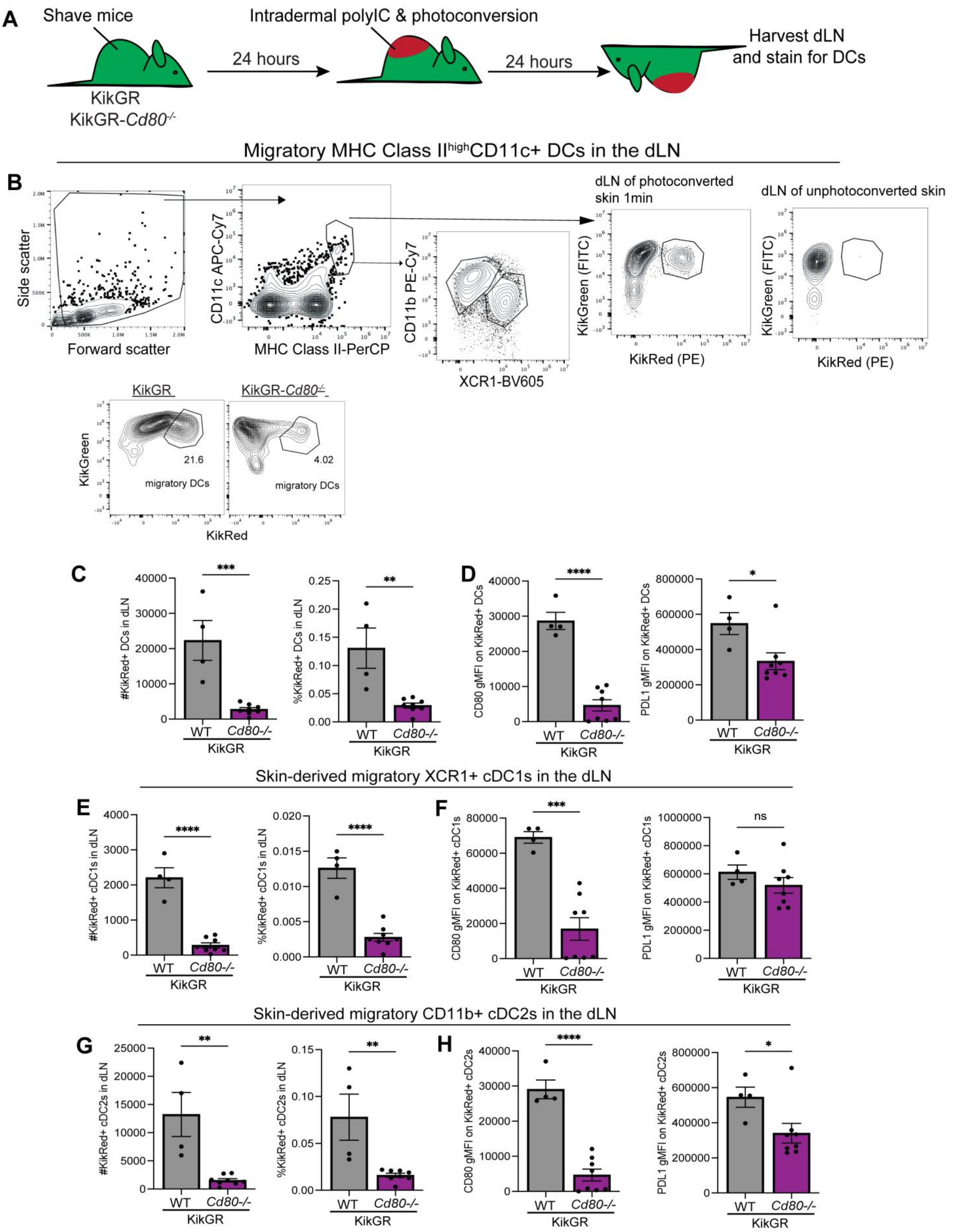
A
Shave mice
KikGR
KikGR-Cd80-/-
24 hours
Intradermal polyIC & photoconversion
24 hours
Harvest dLN and stain for DCs
Migratory MHC Class IIhighCD11c+ DCs in the dLN
B
Side scatter
Forward scatter
CD11c APC-Cy7
MHC Class II-PerCP
CD11b PE-Cy7
XCR1-BV605
dLN of photoconverted skin 1min
KikGreen (FITC)
KikRed (PE)
dLN of unphotoconverted skin
KikGR
KikGR-Cd80-/-
KikGreen
KikRed
21.6
4.02
migratory DCs
C
#KikRed+ DCs in dLN
%KikRed+ DCs in dLN
D
CD80 gMFI on KikRed+ DCs
PDL1 gMFI on KikRed+ DCs
Skin-derived migratory XCR1+ cDC1s in the dLN
E
#KikRed+ cDC1s in dLN
%KikRed+ cDC1s in dLN
F
CD80 gMFI on KikRed+ cDC1s
PDL1 gMFI on KikRed+ cDC1s
ns
Skin-derived migratory CD11b+ cDC2s in the dLN
G
#KikRed+ cDC2s in dLN
%KikRed+ cDC2s in dLN
H
CD80 gMFI on KikRed+ cDC2s
PDL1 gMFI on KikRed+ cDC2s
WT
Cd80-/-
KikGR

S3. KikGR mice treated with 10F9.G2 and 43H12 antibodies have fewer KikRed+ DCs and less PD-L1 and CD80 surface expression in the dLN 24 hours after polyI:C injection.
A) Experimental scheme for Figure S4 B-H.
B) Gating strategy for KikGR DCs. Representative flow cytometry plots showing migratory KikRed+ DCs in the dLN 24 hours after intradermal polyI:C injection.
C) Number and percentage of KikRed+ DCs (combined cDC1s and cDC2s) in the dLN 24 hours after intradermal polyI:C injection.
D) Surface expression of CD80 and PD-L1 evaluated by geometric mean fluorescence intensity (gMFI) on FITC+ DCs in the dLN 24 hours after intradermal polyI:C injection
E) Number and percentage of KikRed+ cDC1s in the dLN 24 hours after intradermal polyI:C injection.
F) Surface expression of PD-L1 and CD80 evaluated by gMFI on FITC+ cDC1s in the dLN 24 hours after intradermal polyI:C injection
G) Number and percentage of KikRed+ cDC2s in the dLN 24 hours after intradermal polyI:C injection.
H) Surface expression of PD-L1 and CD80 evaluated by gMFI on FITC+ cDC2s in the dLN 24 hours after intradermal polyI:C injection

Data shown is representative of one experiment. Each dot represents one lymph node where at least 2 mice per group were used. Experiment was completed on 2 separate occasions with 2-4 mice per treatment group. Similar results were acquired in the second experiment. Statistical significance was determined using student's t-test. *$p<0.05$, **$p<0.01$, ****$p<0.0001$; n.s., $p>0.05$. Error bars indicate standard error of the mean.

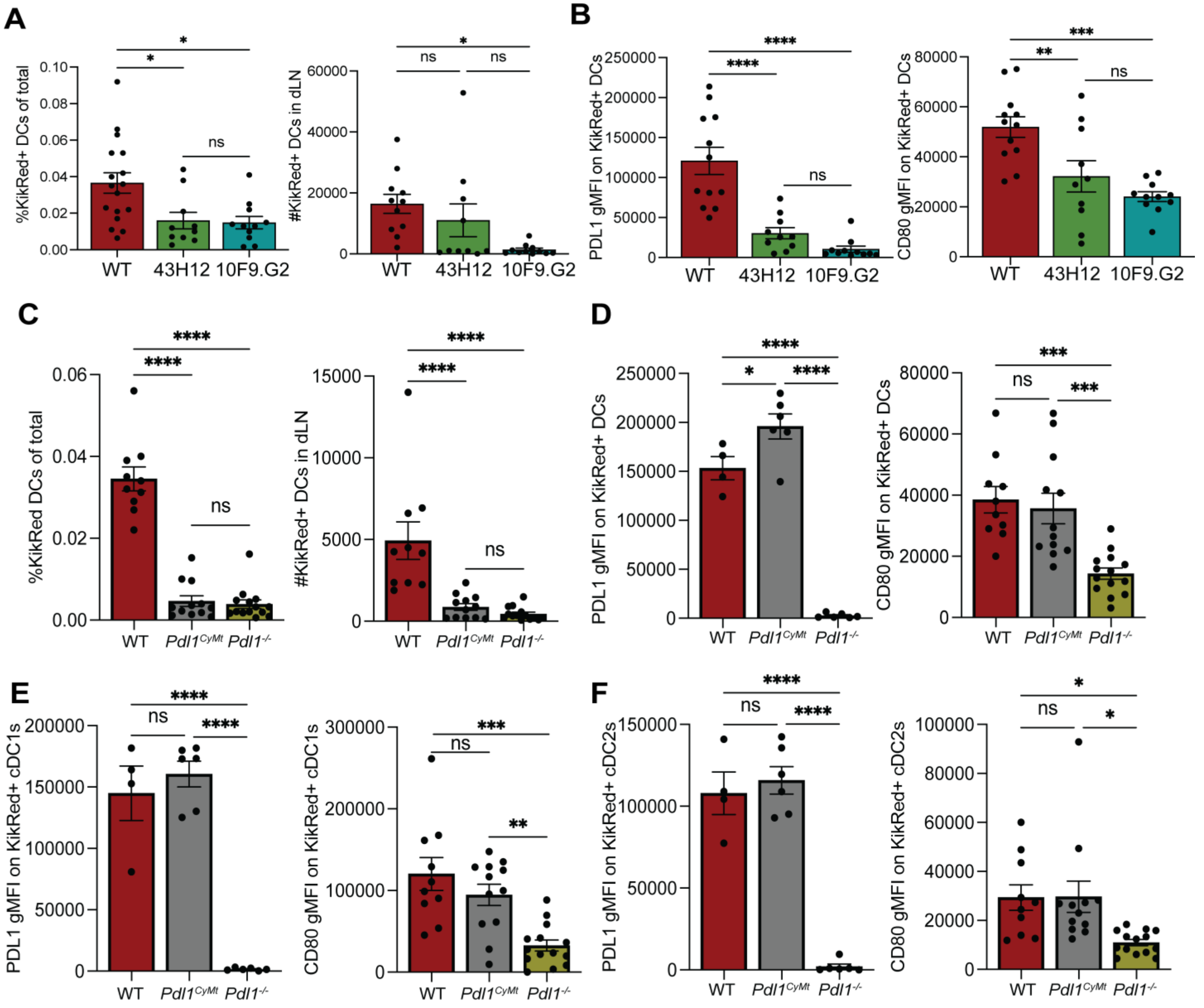

S4. KikGR mice treated with PD-L1 and PD-1 antibodies have differences in amounts of KikRed+ DCs in the dLN 24 hours after polyI:C injection.

A) Number and percentage of KikRed+ DCs (combined cDC1s and cDC2s) in the dLN 24 hours after intradermal polyI:C injection and indicated antibody.

B) Surface expression of PD-L1 and CD80 evaluated by geometric mean fluorescence intensity (gMFI) on KikGR+ DCs in the dLN 24 hours after intradermal polyI:C injection and indicated antibody.

C) Percent and number of KikRed+ DCs in dLN 24 hours after intradermal polyI:C injection in WT, *Pdl1-/-* and *Pdl1^{CyMt}* mice.

D) Surface expression of PD-L1 and CD80 evaluated by gMFI on KikGR+ DCs (cDC1 and cDC2) in the dLN 24 hours after intradermal polyI:C injection.

E) Number and percentage of KikRed+ cDC1s in the dLN 24 hours after intradermal polyI:C injection.

F) Surface expression of PD-L1 and CD80 evaluated by geometric mean fluorescence intensity (gMFI) on KikRed+ cDC2s in the dLN 24 hours after intradermal polyI:C injection

Data shown in figure represents 2 combined experiments. Each dot represents one lymph node where 2-5 mice per group were used. Experiment was completed on 3-5 separate occasions. For G-H PD-L1 MFI is only shown from one experiment due to the use of a different fluorophore in the repeat experiment being of different intensity. Experimental repeat with different fluorophore was still not different between WT and *Pdl1^{CyMt}* and no staining was evident in *Pdl1-/-*. Statistical significance was determined using student's t-test. *p<0.05, **p<0.01, ****p<0.0001; n.s., p>0.05. Error bars indicate standard error of the mean.

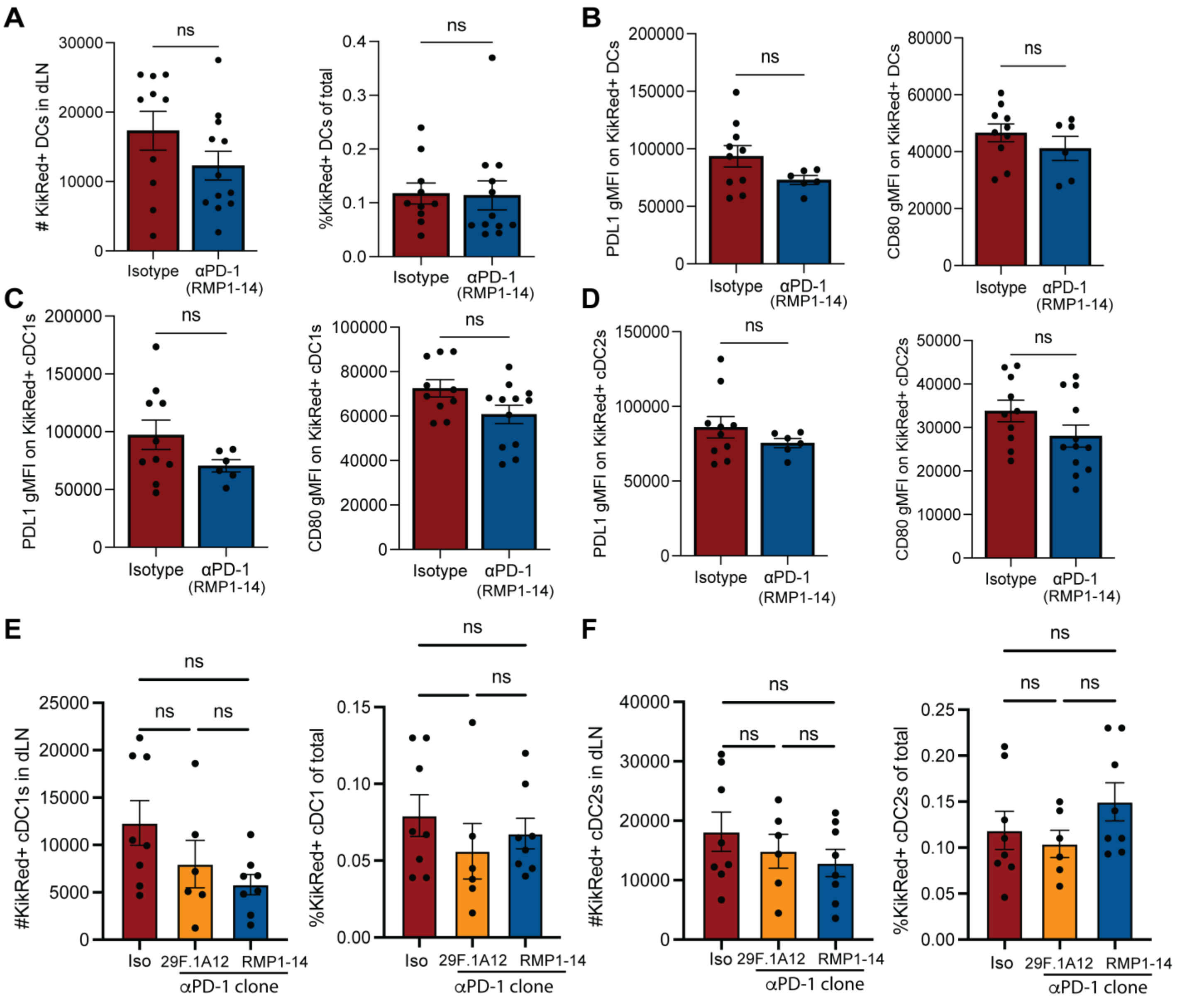

S5. Anti-PD-1 clones do not decrease DC migration or surface PD-L1 and CD80 levels.

A) Number and percent of total of KikRed positive total DCs 24 hours after intradermal polyI:C in draining LN of mice treated with isotype or RMP1-14 anti-PD-1 clone (200μg) 24 hours before and on day of conversion.

B) PD-L1 and CD80 gMFI of all migrated DCs from A.

C) PD-L1 and CD80 gMFI of migrated cDC1s in dLN.

D) PD-L1 and CD80 gMFI of migrated cDC2s in dLN.

E) As in A except a separate group of mice were treated with the 29F.1A12 clone of anti-PD-1. Shown are the number and percent of cDC1s in draining LN that were KikRed positive.

F) As in E except for cDC2s.

Data shown in figure is representative of 2-3 independent experiments with 3-6 replicates per treatment group. Statistical significance was determined using unpaired student’s t-test or one-way ANOVA. n.s., $p>0.05$. Error bars indicate standard error of the mean.

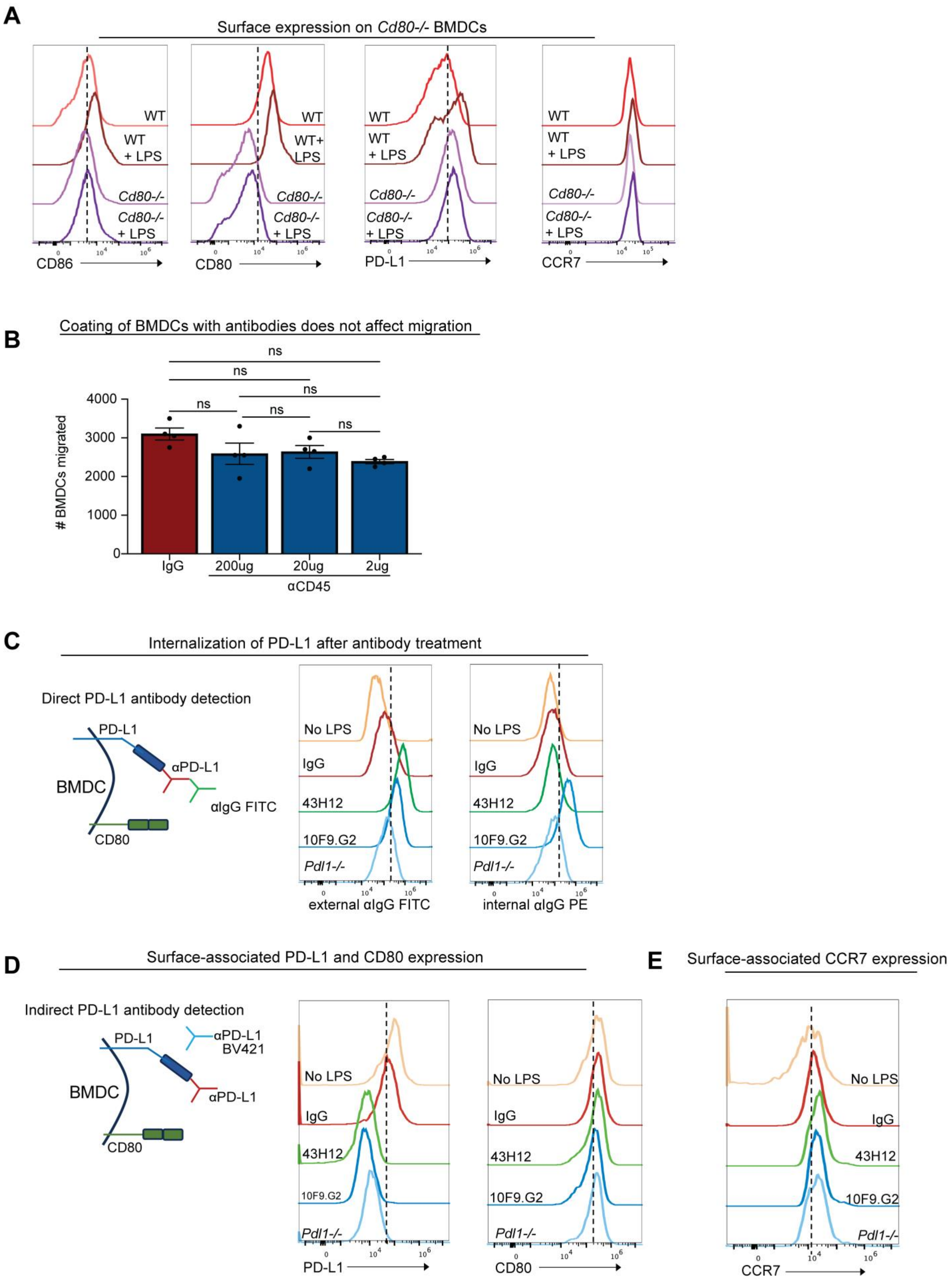
A
Surface expression on Cd80-/- BMDCs
WT
WT + LPS
Cd80-/-
Cd80-/- + LPS
CD86
WT
WT+ LPS
Cd80-/-
Cd80-/- + LPS
CD80
WT
WT + LPS
Cd80-/-
Cd80-/- + LPS
PD-L1
WT
WT + LPS
Cd80-/-
Cd80-/- + LPS
CCR7
B
Coating of BMDCs with antibodies does not affect migration
ns
ns
ns
ns
ns
ns
BMDCs migrated
4000
3000
2000
1000
0
IgG
200ug
20ug
2ug
αCD45
C
Internalization of PD-L1 after antibody treatment
Direct PD-L1 antibody detection
PD-L1
αPD-L1
BMDC
αIgG FITC
CD80
No LPS
IgG
43H12
10F9.G2
Pdl1-/-
external αIgG FITC
No LPS
IgG
43H12
10F9.G2
Pdl1-/-
internal αIgG PE
D
Surface-associated PD-L1 and CD80 expression
Indirect PD-L1 antibody detection
PD-L1
αPD-L1 BV421
BMDC
αPD-L1
CD80
No LPS
IgG
43H12
10F9.G2
Pdl1-/-
PD-L1
No LPS
IgG
43H12
10F9.G2
Pdl1-/-
CD80
E
Surface-associated CCR7 expression
No LPS
IgG
43H12
10F9.G2
Pdl1-/-
CCR7

S6. Surface-associated PD-L1, CD80 and CCR7 expression on DCs.

A) Surface expression of CD86, CD80, PD-L1 and CCR7 on GM-CSF-derived wildtype or *Cd80-/-* BMDCs 4 hours after LPS stimulation. Representative flow plots of 3 independent experiments.

B) Number of migrated GM-CSF-derived BMDCs across a 5μm transwell membrane after 4 hours. BMDCs prior to transwell were treated with either 2, 20, or 200μg/mL of CD45 antibody and LPS (2ng/mL).

C) Detection of PD-L1-bound antibodies with anti-IgG antibodies on the cell surface and internally. Representative flow plots of 3 independent experiments.

D) Flow plots displaying PD-L1 and CD80 surface expression using either a PD-L1 directly conjugated flow antibody or CD80 directly conjugated antibodies on LPS-activated BMDCs. Data are shown with n=3 replicates per group.

E) Surface expression of CCR7 on LPS-activated BMDCs that were treated with IgG, 10F.9G2, and 43H12 antibodies for 4 hours.

Data shown in figure is representative of 2 independent experiments with 1-3 replicates per treatment group. Statistical significance was determined using student's t-test or one-way ANOVA. *$p<0.05$, **$p<0.01$, ****$p<0.0001$; n.s., $p>0.05$. Error bars indicate standard error of the mean.

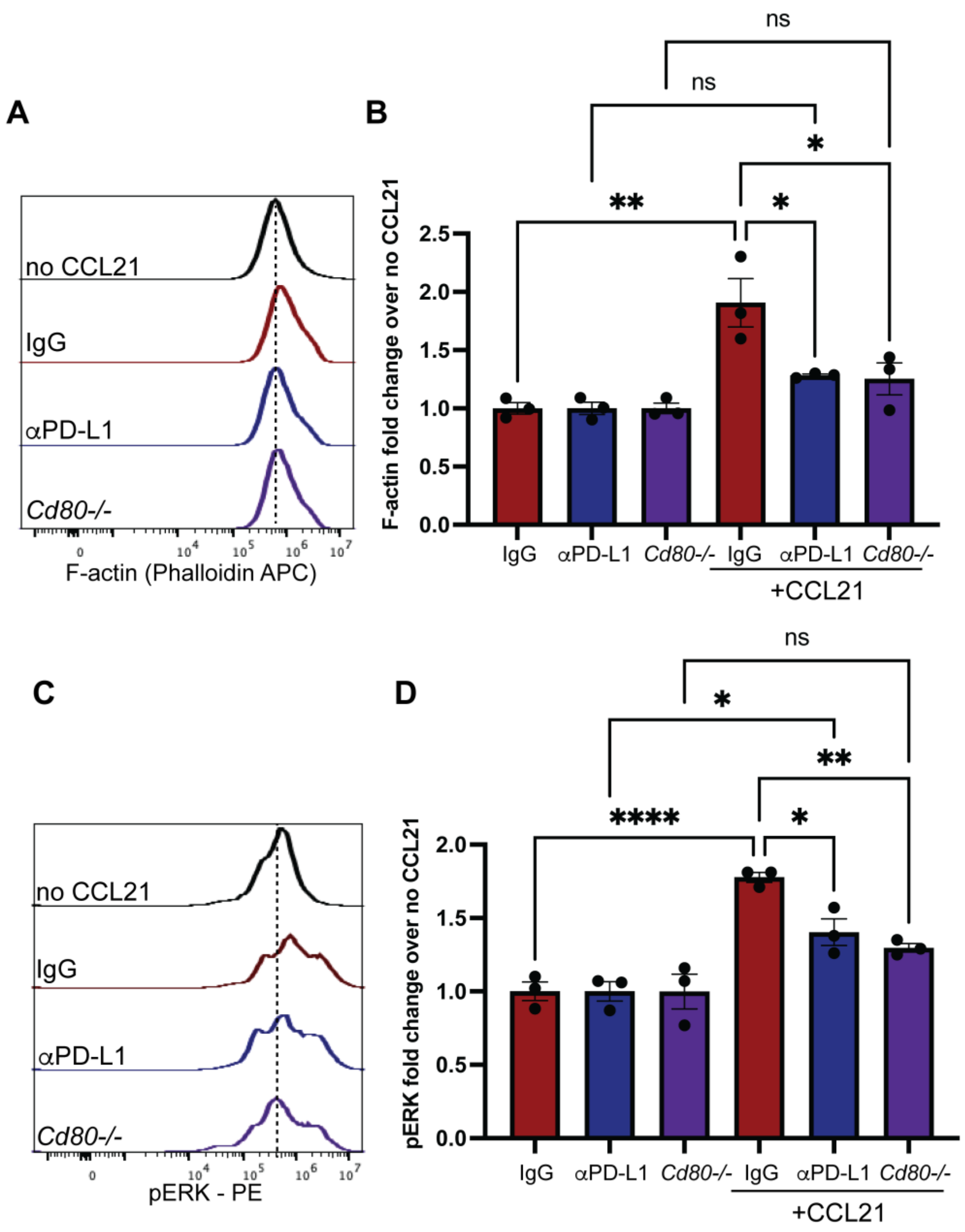

S7. PD-L1 antibody and *Cd80-/-* DCs fail to polymerize actin or phosphorylate ERK.
A) Example flow plot for F-actin for each group.
B) Fold change in MFI of F-actin after phalloidin staining on CCL21-pulsed BMDCs that were treated with either IgG, αPD-L1 10F9.G2, or *Cd80-/-* BMDCs for 4 hours.
C) Example flow plot for phosphor-ERK staining for each group.
D) Fold change in MFI of phosphor-ERK on CCL21-pulsed BMDCs that were treated with either IgG, αPD-L1 10F9.G2, or *Cd80-/-* BMDCs for 4 hours.

Data shown in figure is representative of 2 independent experiments with 1-3 replicates per treatment group. Statistical significance was determined using student's t-test or one-way ANOVA. *$p<0.05$, **$p<0.01$, ****$p<0.0001$; n.s., $p>0.05$. Error bars indicate standard error of the mean.

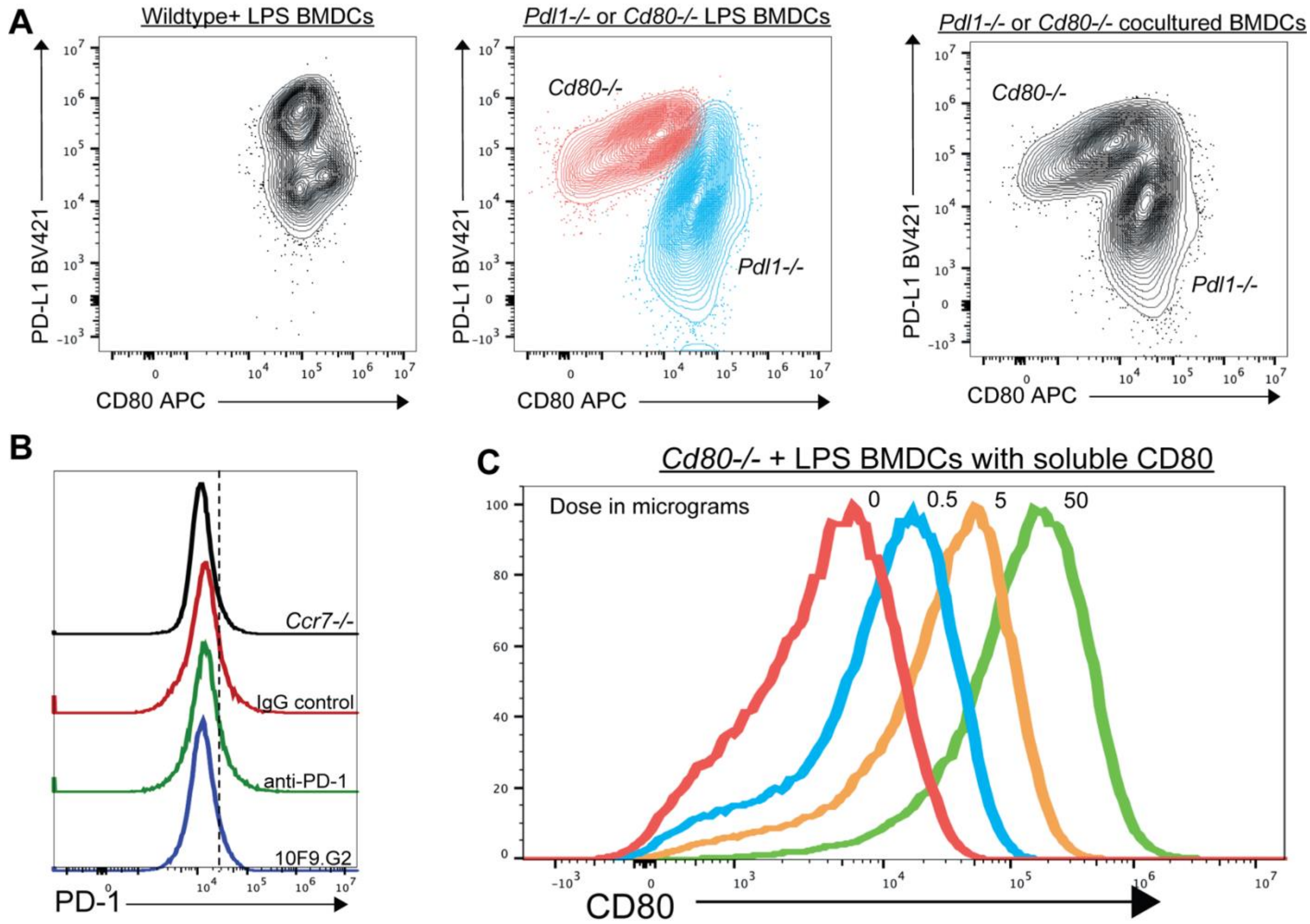

S8. Murine BMDCs do not express appreciable levels of PD-1 and treatment of *Cd80-/-* BMDCs with varying doses of sCD80.

A. Representative flow plots of GM-CSF derived BMDCs after 4 hours of LPS treatment. Gated on MHC Class II high, CD11c high cells. *Pdl1-/-* BMDCs and *Cd80-/-* BMDCs were either independently cultured or cocultured for 4 hours prior to flow cytometry.

B. Surface expression of PD-1 on GM-CSF-derived wildtype or *Ccr7-/-* BMDCs 4 hours after LPS stimulation and 10F9.G2 or 43H12 PD-L1 antibody treatment. Representative flow plots of 3 independent experiments.

C. CD80 surface expression on Cd80-/-BMDCs treated with varying doses of soluble CD80 (sCD80) for 4 hours with simultaneous LPS activation. The doses were 0.5, 5 or 50 μg/mL.

Data shown in figure is representative of 2 independent experiments with 1 technical replicate per treatment group.

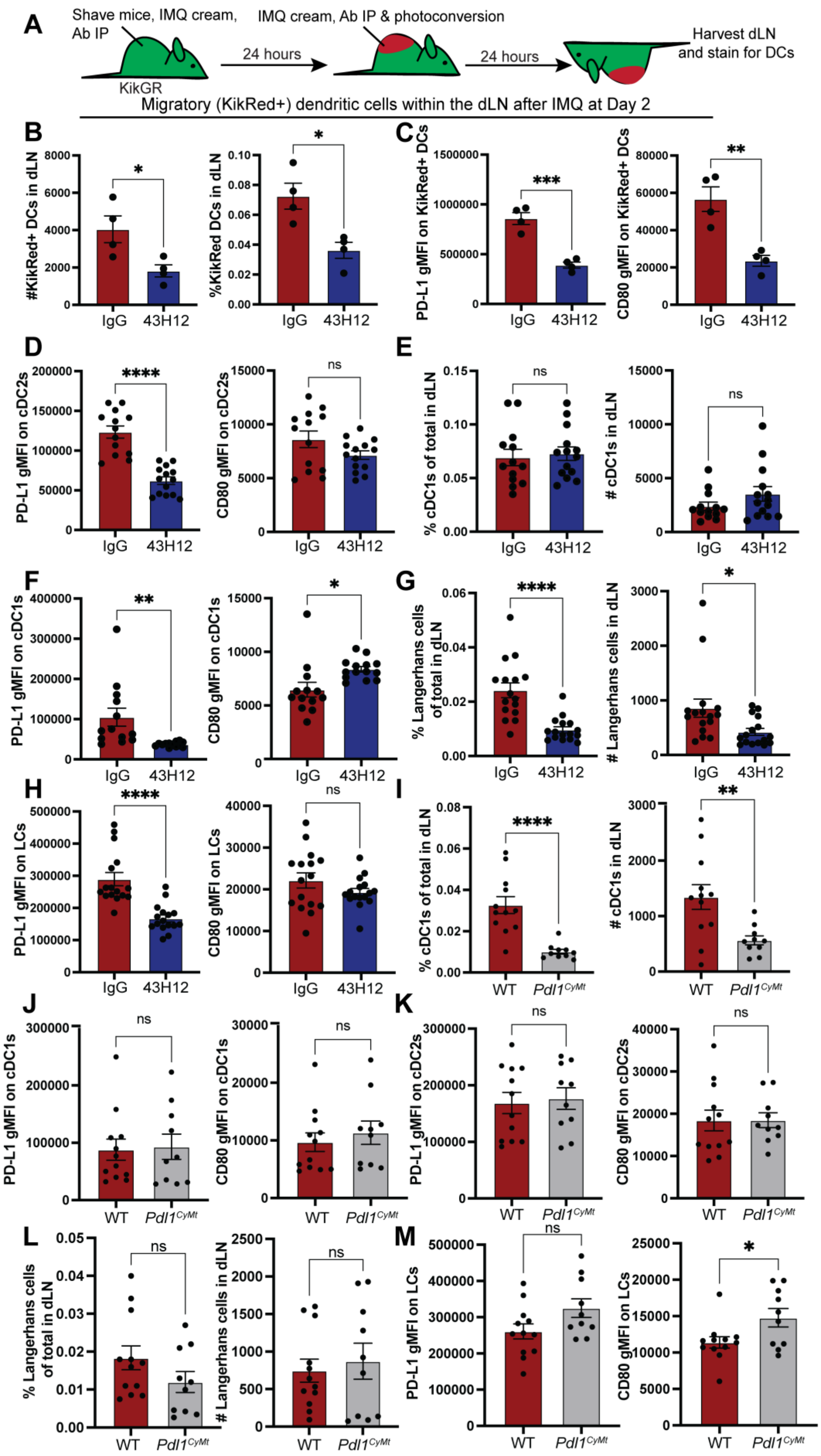
A
Shave mice, IMQ cream, Ab IP
KikGR
24 hours
IMQ cream, Ab IP & photoconversion
24 hours
Harvest dLN and stain for DCs
Migratory (KikRed+) dendritic cells within the dLN after IMQ at Day 2
B
#KikRed+ DCs in dLN
%KikRed DCs in dLN
C
PD-L1 gMFI on KikRed+ DCs
CD80 gMFI on KikRed+ DCs
D
PD-L1 gMFI on cDC2s
CD80 gMFI on cDC2s
E
% cDC1s of total in dLN
# cDC1s in dLN
F
PD-L1 gMFI on cDC1s
CD80 gMFI on cDC1s
G
% Langerhans cells of total in dLN
# Langerhans cells in dLN
H
PD-L1 gMFI on LCs
CD80 gMFI on LCs
I
% cDC1s of total in dLN
# cDC1s in dLN
J
PD-L1 gMFI on cDC1s
CD80 gMFI on cDC1s
K
PD-L1 gMFI on cDC2s
CD80 gMFI on cDC2s
L
% Langerhans cells of total in dLN
# Langerhans cells in dLN
M
PD-L1 gMFI on LCs
CD80 gMFI on LCs
IgG
43H12
WT
Pdl1CyMt
ns

S9. Skin-derived dendritic cell subsets in the skin draining lymph after imiquimod treatment.
A) Experimental scheme in S7 B-C
B) Number and percent of KikRed+ DCs in draining lymph node after 2 days of IMQ topically and 200μg 43H12 antibody intraperitoneally.
C) PD-L1 and CD80 gMFI on DCs in the skin-draining lymph node in IgG and 43H12 antibody-treated mice.
D) PD-L1 and CD80 gMFI on cDC2s in the skin-draining lymph node in IG and 43H12 antibody-treated mice.
E) cDC1 percentage and number in the skin-draining lymph node in IgG and 43H12 antibody-treated mice after topical imiquimod treatment. Gated on MHC Class II high, CD11c+, XCR1+ cells
F) PD-L1 and CD80 gMFI on cDC1s in the skin-draining lymph node in IG and 43H12 antibody-treated mice.
G) Langerhans cell (LC) percentage and number in the skin-draining lymph node in IgG and 43H12 antibody-treated mice after topical imiquimod treatment. Gated on MHC Class II high, CD11c+, CD11b+, Langerin+ cells.
H) PD-L1 and CD80 gMFI on Langerhans cells in the skin-draining lymph node in IgG and 43H12 antibody-treated mice.
I) As in E except with WT and $Pdl1^{CyMt}$ mice.
J) As in F except with WT and $Pdl1^{CyMt}$ mice.
K) As in D except with WT and $Pdl1^{CyMt}$ mice.
L) As in G except with WT and $Pdl1^{CyMt}$ mice.
M) As in H except with WT and $Pdl1^{CyMt}$ mice.

Data shown in figure is representative of 2-3 independent experiments with 4-8 replicates per treatment group. Statistical significance was determined using paired student’s t-test. *$p<0.05$, **$p<0.01$, ****$p<0.0001$; n.s., $p>0.05$. Error bars indicate standard error of the mean.

Supplemental Table 1: List of blood donors and associated information.

A.

**Patient number**	**Age at time of blood draw**	**Sex (M/F)**	**Race/Ethnicity**
HC3	35	Male	Caucasian
HC8	40	Male	Caucasian
HC21	33	Male	Asian
HC28	31	Female	Caucasian
HC59	29	Female	Caucasian
HC74	28	Female	Caucasian

B.

**Patient number**	**Age at time of blood draw**	**Sex (M/F)**	**Race/Ethnicity**
3709	37	Male	White
3711	47	Male	African American
3734	35	Male	White
3745	31	Female	White
3689	37	Male	Hispanic
4.001	26	Female	White

A) Blood donor information associated with Figure 1. Donors from Part A were obtained from the Human Immune Tissue Network Biobank (COMIRB # 17-2159) and were collected at the University of Colorado Clinical and Translation Research Centers (CTRC)

B) Blood donor information associated with Figure 1. Donors from Part B were obtained from were selected from a biorepository of patients who had undergone liver transplantation and collected under IRB protocol 06-0566 and 23-2301.

Supplementary Data S1. Excel document displaying numeric values associated with Figures 1-7 and Figures S1-S9 in each tab.
